# GIS-integrated multi-criteria decision framework for waste-to-energy plant site selection in Beni Suef governorate, Egypt

**DOI:** 10.1038/s41598-026-59518-3

**Published:** 2026-06-30

**Authors:** Wael Mostafa, Saif M. Abo Khashaba, Abdelhamid Elsabagh, Zenhom Magd, Hussein Abdelfattah M. Abdelkhalek, Mohamed Rabie Abdelzaher

**Affiliations:** 1 Geography Department, Faculty of Arts, Kafrelsheikh University, Kafrelsheikh, 33516 Egypt; 2 Geology Department, Faculty of Science, Kafrelsheikh University, Kafrelsheikh, 33516 Egypt; 3 Geography Department, Faculty of Arts, Port Said University, Port Said, 42526 Egypt; 4 Geography Department, Faculty of Arts, Beni-Suef University, Beni Suef, 62511 Egypt

**Keywords:** Waste-to-Energy, GIS-MCDM, Analytical Hierarchy Process, Site suitability, Spatial planning, Geological constraints, Municipal solid waste, Environmental sciences, Environmental social sciences, Natural hazards

## Abstract

This study presents the first comprehensive GIS-MCDM site suitability model for a Waste-to-Energy (WTE) facility in Upper Egypt. A sixteen-criterion analytical framework encompassing environmental protection, geological safety, infrastructure accessibility, and social proximity constraints was developed through a structured expert consultation process involving 42 specialists from academic, governmental, and environmental sectors. Criterion weights were derived using the Analytical Hierarchy Process (AHP) and validated with a Consistency Ratio of 2.6% (well below the 10% threshold). Spatial data layers were derived from Landsat-9 imagery (SVM classification), ASTER GDEM (30 m), ERA5-Land wind reanalysis, World population grids, OpenStreetMap infrastructure networks, and the Conoco–EGPC geological map of Egypt. Across the 10,698.5 km² study area, the integrated suitability map reveals that zones classified as high or very high suitability together constitute only 2.02% of the total area (59.5 km²; very high: 0.19%, 6.3 km²; high: 1.83%, 53.2 km²). The dominant land constraint, 69.3% classified as very low suitability, reflects strict environmental exclusion buffers around protected areas (PA; weight 11.4%), sensitive land uses (SU; 11.2%), surface water bodies (SW; 9.4%), and steep terrain (SP; 9.4%). Three candidate sites with high suitability scores were delineated, with the most favorable located east of Beni Suef city (coordinates: 29°01′ N, 31°07′ E; area: 22.75 km²), proximate to the governorate’s largest existing landfill (~ 2.6 km) and with favorable north-westerly wind alignment relative to populated zones. This study advances the GIS-MCDM literature by integrating geological (faults, lithology, soil bearing capacity) and environmental safety criteria within an arid-region planning context, an approach insufficiently addressed in prior Egypt-focused or MENA (WTE) siting studies. The resulting suitability model constitutes a reproducible, evidence-based decision-support tool for Egyptian environmental planners and aligns with Egypt’s Sustainable Development Strategy 2030 goals for renewable energy diversification and circular economy promotion. The selected site shows potential logistical and economic advantages due to its proximity to existing landfill infrastructure and regional road networks; however, these advantages represent spatial screening indicators and require further techno-economic and network-based transport assessment before implementation. Model validation using ROC–AUC analysis confirmed good discriminatory performance, with an AUC of 0.829, overall accuracy of 90.0%, and Kappa coefficient of 0.801.

## Introduction

The global production of municipal solid waste (MSW) surpassed 2.0 billion tons in 2023 and is projected to reach 3.4 billion tons by 2050 under business-as-usual trajectories, representing one of the most critical sustainability challenges of the current era^1^.In low- and middle-income countries, the management deficit is particularly acute: more than 90% of waste is disposed of through open dumping or burning, contaminating soil, groundwater, and air while contributing measurably to greenhouse gas emissions, primarily through uncontrolled landfill methane^2^. These dynamics are compounded in rapidly urbanizing arid-region nations, where land resources are scarce, environmental carrying capacity is low, and infrastructure investments lag behind demographic pressure.

Egypt is among the most severely affected countries in the MENA region, generating approximately 97 million tons of solid waste per year while possessing one of the lowest per-capita formal waste treatment capacities in North Africa^3^. Nationally, only 47% of households are served by formal waste collection, while the remainder resort to environmentally unsafe practices, including roadside dumping (reported in 19.4% of households), canal disposal, and open burning of waste (13.8% of households)^4^. These figures translate into measurable degradation of water quality, soil productivity, and public health, particularly in rural and peri-urban governorates where environmental regulation and enforcement remain limited. Upper Egypt, encompassing 13 governorates along the Nile south of Greater Cairo, is disproportionately affected by higher poverty rates, older infrastructure, and a narrower strip of habitable, productive land flanked by desert.

Waste-to-Energy (WTE) technologies, including mass-burn incineration, gasification, pyrolysis, and anaerobic digestion, have emerged as an established component of integrated solid waste management (ISWM) strategies in developed countries, simultaneously addressing waste volume reduction, land preservation, and renewable energy generation^5^. The global (WTE) market is expanding rapidly, with installed capacity projected to exceed 600 GW by 2030, driven by co-benefits in decarbonization and circular economy frameworks^6^. However, the deployment of (WTE) infrastructure in developing countries, particularly in the MENA region, remains nascent. Key barriers include capital intensity, technical complexity, inadequate feedstock characterization, and critically the absence of science-based spatial planning frameworks for facility siting^7^.

Site selection for (WTE) facilities is a multi-dimensional spatial problem requiring the simultaneous optimization of environmental protection objectives (minimizing risks to water, ecosystems, and human health), economic efficiency goals (minimizing transport and connection costs), engineering feasibility constraints (stable geology, suitable terrain), and social acceptance considerations (setback distances from residential and sensitive zones)^8^. The complexity and inherent trade-offs among these objectives make (WTE) siting well-suited to Geographic Information Systems (GIS) integrated with Multi-Criteria Decision Making (MCDM) methodologies. GIS provides the spatial analytical framework for overlaying heterogeneous environmental, infrastructural, and demographic datasets, while MCDM techniques, particularly the Analytic Hierarchy Process (AHP), provide a structured, reproducible mechanism for eliciting and operationalizing expert judgments regarding criterion importance^9^.

The GIS-AHP paradigm has been applied to (WTE) and hazardous facility siting in a range of international contexts, including Turkey^10^, Thailand^11^, Oman^12^, Iran^13^, and South Africa^14^, with broadly consistent findings that environmental protection and social proximity criteria carry the highest relative importance weights across expert panels. In Egypt and the broader North Africa region, GIS-MCDM applications for waste facility siting have emerged more recently, with studies addressing Alexandria^15^, the Nile Delta^16,17^, and Cairo’s metropolitan fringe^18^. However, these studies have been geographically concentrated in northern Egypt and have not systematically addressed the arid interior of Upper Egypt, where distinct environmental, geological, and infrastructural conditions substantially alter the weighting and spatial configuration of suitability criteria.

Several critical gaps persist in the (WTE) siting literature as applied to Egypt and comparable arid-region developing countries. First, prior GIS-AHP studies in Egypt have largely relied on fewer than 12 criteria, often omitting geological hazard parameters (active-fault proximity, lithological stability, soil bearing capacity) that are particularly important in the tectonically active Eastern Desert margin environment. Second, the remote sensing data inputs in previous work have been primarily limited to land cover classification, without systematic integration of terrain analysis (slope, aspect) or wind climate data for emission dispersion assessment. Third, few studies have explicitly addressed the challenge of sparse suitable land availability in arid governorates, where the combination of agricultural land protection and environmental exclusion zones severely constrains feasible development footprints.

This study addresses these gaps by presenting a comprehensive, sixteen-criterion GIS-AHP suitability assessment for (WTE) facility siting in Beni Suef Governorate, Upper Egypt, a region that simultaneously exemplifies the waste management crisis of Upper Egypt and offers a geographically diverse landscape (Nile floodplain, desert plateau, fault-dissected escarpments) that tests the full range of siting criteria. The analytical framework integrates satellite remote sensing Landsat-9 SVM land cover classification, ASTER GDEM terrain analysis), ERA5-Land wind climate reanalysis, WorldPop demographic gridding, and geoscientific datasets (geological map, fault traces, soil surveys) within ArcGIS Pro. An expert consultation panel of 42 specialists from academic, governmental, and environmental management sectors provided criterion importance ratings, which were formalized into AHP weights validated by a Consistency Ratio of 2.6%. The resulting suitability model is the first of its kind in Upper Egypt and provides an actionable, reproducible spatial decision-support tool for Egyptian environmental planners and waste management authorities.

The specific objectives of this study are: (i) to develop a locally adaptive sixteen-criterion suitability framework for (WTE) facility siting in an arid Upper Egyptian context; (ii) to produce a spatially explicit, quantitative suitability map for Beni Suef Governorate at 30 m spatial resolution; (iii) to identify and characterize the most suitable candidate sites; and (iv) to contextualize the results within the national and regional GIS-MCDM literature, highlighting the contribution of geological and environmental criteria to siting outcomes in arid regions.

## Study area

**Fig. 1:**
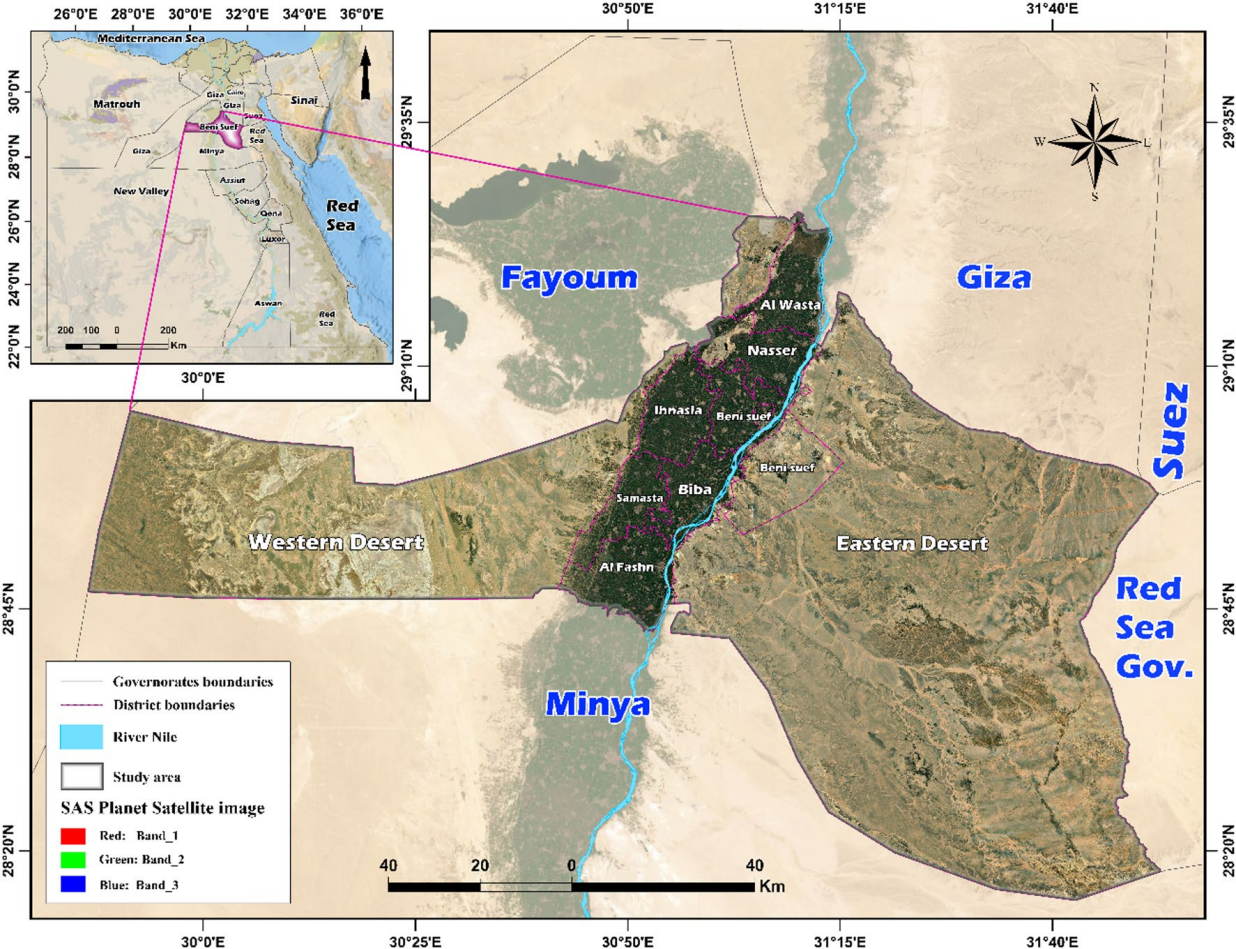
Location of Beni Suef Governorate (the red lines delineate the boundaries of the administrative districts^21^. This figure was created by ArcGIS Pro version 3.6. https://www.esri.com/en-us/arcgis/products/arcgis-pro/resources.

Beni Suef Governorate is situated in the central stretch of the Nile Valley, constituting a pivotal geographic nexus between Lower Egypt (the Delta region) and the southern governorates of Upper Egypt (Fig. 1). The governorate extends between latitudes 28°35′ N and 29°20′ N, and longitudes 30°45′ E and 31°35′ E, spanning a total area of 10,698.5 km² along approximately 110 km of Nile corridor. Administratively, the governorate comprises seven districts: Beni Suef, Al-Wasta, Nasser, Ihnasia, Biba, Al-Fashn, and Sumasta.

Topographically, the governorate encompasses a pronounced east–west gradient: the Nile floodplain and delta fringe attain elevations of approximately 22 m above sea level, while the Eastern Desert plateau rises to more than 350 m, with an intervening escarpment characterized by incised wadis and exposed Eocene–Cretaceous carbonate and clastic formations. The geological framework is dominated by Eocene limestone and chalk in the eastern desert, Quaternary alluvial deposits in the Nile floodplain, and localized exposures of older Nubian Sandstone to the south. Active fault systems, predominantly NW–SE and NE–SW trending, intersect the eastern desert margin and carry implications for geotechnical site stability that are explicitly addressed in the siting framework of this study.

According to national statistics from the Central Agency for Public Mobilization and Statistics^19^, municipal solid waste management in Egypt faces serious challenges, as only about 47% of households depend on formal waste collection services, while the remainder rely on environmentally unsafe disposal practices. Approximately 19.4% of households dispose of waste in canals and drains, and 13.8% burn their waste, contributing to surface-water and air pollution. These figures highlight the urgent need for sustainable Waste-to-Energy (WTE) solutions, particularly in Upper Egypt.

**Fig. 2:**
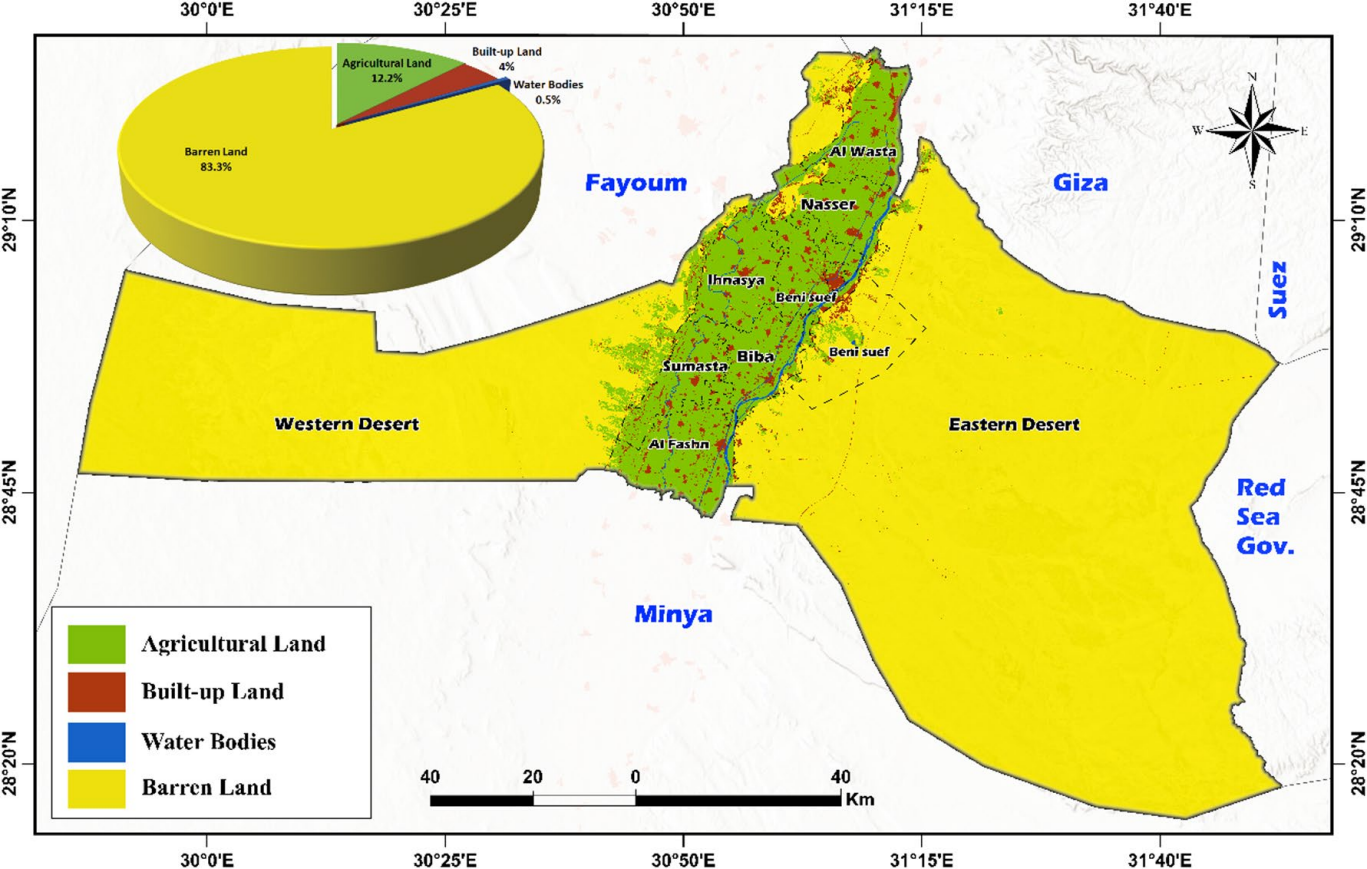
Land cover map of Beni Suef Governorate generated using SVM classification algorithm applied to satellite imagery from July 2025, obtained from Earth Explorer (https://earthexplorer.usgs.gov/). This figure was created by ArcGIS Pro version 3.6. https://www.esri.com/en-us/arcgis/products/arcgis-pro/resources.

**Fig. 3:**
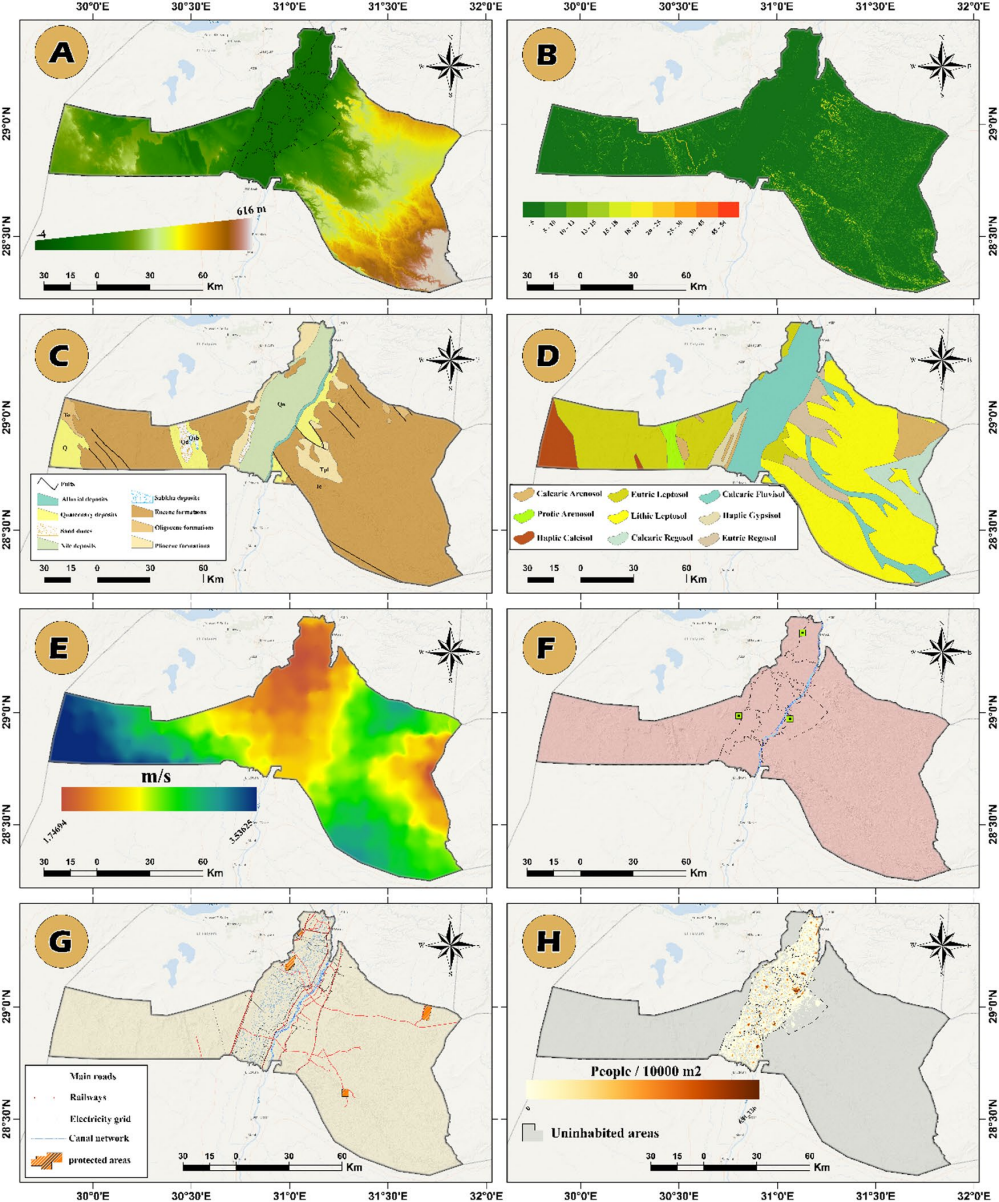
(a) Elevation; (b) Slope; (c) Geology, Faults; (d) Soil; (e) Wind; (f) Landfill, River; (g) Main Roads, Electricity Grid, Railways, canals; (h) People in the study area of Beni Suef Governorate. This figure was created by ArcGIS Pro version 3.6. https://www.esri.com/en-us/arcgis/products/arcgis-pro/resources.

In this context, Beni Suef Governorate represents a critical case study area. According to the Central Agency for Public Mobilization and Statistics^4^ Beni Suef has a population of approximately 3,979,422 inhabitants and covers an area of about 10,698.5 km² (Table 1; Fig. 2). The governorate is characterized by an arid-to-semiarid climate, with very low annual rainfall of 10–20 mm, and prevailing winds predominantly from the north and northwest, particularly during the summer months (Fig. 3E). These geographical and climatic characteristics increase the environmental risks associated with improper waste disposal and strengthen the importance of applying integrated and sustainable (WTE) planning strategies in the region.

**Table 1:** Land classification of Beni Suef Governorate performed by the General Authority for Urban Planning using the Support Vector Machine (SVM) algorithm (https://earthexplorer.usgs.gov/).

**Classification**	**Area (km)²**	**Percentage (%)**
Agricultural lands	1306.6	12.2
Land availability (barren land)	8911	83.3
Built-up land	429.3	4
Water bodies	51.6	0.5
**Total**	**10698.5**	**100**

Agriculture represents one of the main economic activities in Beni Suef, with major crops including wheat, maize, sugar beet, cotton, and vegetables^20^. Industrial zones such as Beni Suef Industrial Zone and Kom Abu Radi Industrial Complex contribute significantly to local waste generation. The governorate also includes important archaeological and heritage sites, such as the Medium Pyramid, monasteries, and Pharaonic remains, reflecting diverse socioeconomic and cultural characteristics.

A land cover classification was conducted using the Support Vector Machine (SVM) algorithm, which is known for its high accuracy in processing multispectral satellite imagery. The SVM classifier successfully distinguished the major land cover classes across Beni Suef Governorate. The results indicate that agricultural lands occupy approximately 12.2% of the governorate, while urban areas cover about 4%. Water bodies, mainly the Nile River, account for around 0.5%, whereas vacant and unused lands represent the largest share with nearly 83.3% of the total area. These spatial distributions are illustrated in Fig. 2.

## Production of solid waste

Solid waste refers to discarded solid materials generated from residential, commercial, industrial, and agricultural activities. Proper solid waste management is a fundamental component of environmental protection and sustainable development, particularly in rapidly growing urban regions^17^.

Beni Suef Governorate has experienced substantial demographic growth over recent decades, a trend that is projected to continue in the coming years. As presented in Table 2, the total population increased from approximately 2.29 million in 2006 to 3.98 million in 2025 and is expected to exceed 5.32 million by 2035. This demographic expansion is unevenly distributed across districts, with Beni Suef, Al Wasta, and Biba exhibiting the most pronounced population increases.

This rapid population growth has been accompanied by a significant rise in solid waste generation. The total quantity of municipal solid waste increased from 455.77 tons/day in 2020 to 521.69 tons/day in 2025 and is projected to reach approximately 695.69 tons/day by 2035, representing an increase of more than 52% over 15 years. Among the districts, Beni Suef City generates the highest share of waste, followed by Al Wasta and Sumasta, reflecting their population sizes and urban activity levels.

**Table 2:** The latest Population 2006–2025^19^, Projected Population on 2030–2035. (SWM, Beni Suef Governorate 2025), Projected SW in 2030–2035. Abbreviations: Population (Pop.), Quantity of Waste (Q.W); Expected Quantity of Waste (E.Q.W).

District	Pop. 2006	Pop.2020	Pop.2025	Pop.2030	Pop. 2035	Q.W 2020 (tons/day)	Q.W 2025 (tons/day)	E.Q.W 2030 (tons/day)	E.Q.W 2035 (tons/day)
Beni Suef	509,106	750,781	862,513	990,871	1,138,333	120.36	136.62	156.96	180.32
Nasser	358,741	403,648	463,823	532,968	612,422	17.77	37.92	43.57	50.07
Al Wasta	273,542	534,851	616,851	711,422	820,492	108.40	121.31	139.91	161.36
Ihnasiya	283,514	425,096	491,262	567,727	656,093	62.77	61.18	70.70	81.70
Biba	329,234	513,804	602,324	706,095	827,744	43.00	46.75	54.81	64.25
Al Fashn	337,132	512,283	594,849	690,722	802,047	28.57	34.91	40.54	47.07
Sumasta	200,439	300,845	347,800	402,083	464,838	74.90	83.00	95.95	110.92
Total	2,291,708	3,441,308	3,979,422	4,601,888	5,321,969	455.77	521.69	602.43	695.69

The observed relationship between population growth and solid waste generation demonstrates a strong positive correlation, indicating that demographic expansion remains a primary driver of waste production in the governorate. If current trends persist, the anticipated increase in waste quantities will place considerable pressure on existing waste management infrastructure and disposal facilities.

Consequently, these projections underscore the urgent need for sustainable and efficient waste management solutions, particularly the adoption of waste-to-energy (WTE) technologies. Strategic planning for (WTE) facilities requires accurate estimation of future waste availability and spatial distribution, which forms a critical foundation for the site suitability analysis conducted in this study using GIS and remote sensing techniques.

## Methodology

### Choosing decision levels (objectives, standards) and practical considerations

The siting of Waste-to-Energy (WTE) facilities represents a critical planning challenge, as it requires balancing environmental protection, economic viability, technical feasibility, and social acceptance. International experience demonstrates that although these core objectives are widely shared, decision-making standards vary considerably across countries and even between regions within the same country due to heterogeneity in physical, environmental, and socio-economic conditions^22^.

In Beni Suef Governorate (Upper Egypt), rapid population growth and accelerating municipal solid waste generation have heightened the need for integrated, scientifically robust waste management strategies. The primary objective of this study is to identify optimal sites for (WTE) facility development that minimize adverse environmental impacts, preserve natural and agricultural resources, and reduce infrastructure and operational costs while satisfying engineering and logistical constraints.

Unlike several countries with explicit regulatory frameworks governing (WTE) site selection and environmental impact assessment, Egypt currently lacks specific, binding national criteria dedicated to the spatial planning of (WTE) facilities. To address this gap, this study developed a locally adaptive, multi-criteria decision-making framework based on a critical review of international best practices, detailed analysis of the environmental and infrastructural characteristics of Beni Suef Governorate, and structured consultation with subject-matter experts.

Sixteen sub-criteria were systematically classified and grouped into environmental, technical, economic, and social domains. The relative importance of these criteria was quantified through a structured expert-based questionnaire using a 1–9 scale within the Analytical Hierarchy Process (AHP) framework^9^. Expert participants represented academic institutions and governmental sectors, including housing, utilities, urban planning, environmental protection, electricity, and energy authorities. This integrative approach ensured that the final site suitability model reflects both scientific rigor and practical decision-making relevance.

It should be noted that the environmental component of this study represents a spatial environmental screening stage rather than a full project-level Environmental Impact Assessment (EIA). The adopted criteria, including distance from sensitive land uses, surface water, protected areas, agricultural land, population-density zones, and prevailing wind conditions, are intended to minimize potential environmental and social exposure at the site-selection stage. However, detailed EIA procedures, including stack-emission modelling, air-dispersion analysis, ash-management assessment, groundwater vulnerability analysis, traffic impact assessment, and public-health risk evaluation, remain necessary before final site approval and implementation.

#### Distance to sensitive land uses

Mitigating noise, odor, and emissions from Waste-to-Energy (WTE) plants during operation remains a significant challenge due to their adverse effects on human health. Therefore, site selection must prioritize locating these facilities at a safe distance from densely populated areas and key tourist destinations within Beni Suef Governorate^23,24^.

Increasing the buffer distance between the plant and sensitive land uses such as residential neighborhoods, hospitals, schools, tourist sites, commercial areas, mosques, and military zones is essential to minimize environmental and public health impacts. Additionally, future urban development zones are considered in the site selection process to ensure sustainable spatial planning.

Spatial analysis indicates that sensitive land uses constitute approximately 4% of the total area of the study region in Beni Suef (Table 1). Based on prior research, a minimum buffer zone of 500 m around these sensitive areas is recommended within the governorate^23–25^.

#### Distance from agricultural land

The Waste-to-Energy (WTE) plant site should be located away from agricultural lands to prevent contamination of crops and edible plants. Increasing the distance between the facility and agricultural areas enhances site suitability by reducing potential pollution risks. Maintaining an appropriate buffer zone is especially critical given that agricultural lands constitute approximately 12.2% of the total area in Beni Suef Governorate.

Based on an extensive review of the literature, a minimum buffer zone of 500 m is recommended around agricultural lands to safeguard crop safety and environmental quality^25^.

#### Distance to surface water

This criterion is vital from an environmental protection standpoint, as it excludes sites located within 1,000 m of surface water bodies to prevent contamination risks^26^. Surface water features represent approximately 0.5% of the total area of Beni Suef Governorate. Establishing this buffer zone is essential to safeguard water quality and maintain the ecological integrity of the region’s aquatic systems.

#### Distance to landfills

The proximity of Waste-to-Energy (WTE) plants to existing landfills plays a critical role in both economic and environmental aspects of integrated solid waste management, particularly in terms of fuel consumption and pollution generated during waste transportation^24^. Transportation costs significantly influence the operational expenses of (WTE) facilities, directly impacting on their economic feasibility.

To minimize these costs and associated environmental impacts, it is essential to locate (WTE) plants near substantial and consistent sources of solid waste. In Beni Suef Governorate, where there are six active landfills, siting the plant close to these waste disposal sites enhances suitability by reducing transport distances, thereby improving both economic and environmental performance^23^.

#### Distance to the electricity network

The facility should be located close to the existing electricity network to facilitate the integration of the generated power into the grid. In Beni Suef Governorate, the electricity network spans approximately 1,137.8 km, underscoring the importance of proximity to this infrastructure for efficient, cost-effective grid connection^24,27^.

#### Distance to the road network

Selecting a site close to major roadways is a fundamental requirement for WTE facility planning, as transportation of solid waste typically accounts for a substantial share of total operational costs and has a direct bearing on economic feasibility^24,27–29^. Ready access to the existing road network also removes the need to construct dedicated access roads, which would otherwise add considerable infrastructure expenditure to the project. In Beni Suef Governorate, the primary road network extends approximately 1,309.5 km according to the Egyptian Ministry of Transport (General Authority for Roads, Bridges, and Land Transport), offering broad connectivity across the governorate that can support efficient waste collection and haulage operations. At the same time, locating a WTE facility immediately adjacent to major roads raises legitimate concerns about visual intrusion, odor exposure, and traffic safety. For this reason, a minimum standoff distance of 300 m from major roads was adopted as an exclusion threshold. Beyond this buffer, suitability was treated as a function of distance: locations in close operational reach of the road network received higher scores, while those at excessive distances were penalised to reflect the increased cost and complexity of providing adequate road access.

#### Distance to protected areas

Protected areas represent some of the most ecologically sensitive land in any region, and their proximity to industrial facilities has long been a source of concern in environmental planning. In Beni Suef Governorate, such areas cover approximately 87.98 km², and a minimum buffer of 500 m was maintained around their boundaries throughout the siting process to guard against disturbance to habitats and wildlife^15,25^. Establishing the spatial extent of these zones required careful attention to data quality. Boundary delineation relied on authoritative cartographic and institutional reference sources, with topographic maps, OpenStreetMap layers, and ArcGIS basemap imagery used only for supplementary cross-checking and visual verification rather than as primary inputs.

####  Population

Avoiding areas with high population density is a fundamental criterion in selecting the site for a Waste-to-Energy (WTE) facility. This approach helps minimize environmental and social impacts while ensuring greater community acceptance of the project, as illustrated in Table 2; Fig. 3h.

#### Distance to railway line

Although the railway network within Beni Suef Governorate is relatively limited in length, it is essential to maintain a buffer zone of 300 m on both sides of the railway centerline to mitigate the adverse effects of odors and emissions and to preserve the aesthetic environment. Increasing the distance between the railway lines and the Waste-to-Energy (WTE) facility site enhances suitability by minimizing potential environmental and social impacts. According to the Ministry of Transport (National Authority for Egyptian Railways), the total length of railway lines in Beni Suef Governorate is approximately 211.2 km.

#### Wind direction criterion

Wind direction plays an important role in WTE facility siting because odors and airborne emissions tend to follow prevailing wind patterns toward nearby communities. In Beni Suef Governorate, winds predominantly blow from the west and northwest according to records from the National Meteorological Agency, as shown in Fig. 3e. Because this directional pattern remains broadly consistent across the governorate owing to its latitudinal position and regional wind regime, prevailing direction was treated as a qualitative planning consideration when interpreting the downwind exposure of candidate sites rather than as a spatially varying GIS input.

Wind speed, by contrast, shows meaningful spatial variation across the governorate and was therefore adopted as the quantitative criterion in the suitability model. Areas with higher wind speeds were assigned greater suitability on the basis that stronger winds enhance atmospheric dispersion and reduce the likelihood of odor accumulation near the facility.

#### Elevation

Elevation directly influences fuel efficiency for transportation trucks; therefore, selecting a site at a lower elevation is preferable. Higher altitudes pose greater challenges for access and increase transportation costs. Consequently, locations at higher elevations are assigned lower suitability weights. In Beni Suef Governorate, the elevation ranges from approximately − 4 m to 616 m above sea level, as shown in Fig. 3a.

#### Terrain slope 

Flat terrain is more suitable for establishing a Waste-to-Energy (WTE) plant, as the technical feasibility decreases with increasing slope^24,28^. Terrain slope is also an economic factor, as steeper slopes pose greater challenges for construction and access. In Beni Suef Governorate, terrain slope variations are illustrated in Fig. 3b, highlighting the preference for low-slope areas in site selection.

#### Land availability

WTE facilities require substantial undeveloped land to accommodate construction, operational infrastructure, and potential future expansion. Sites should therefore be located on barren, abandoned, or otherwise unused land, away from areas currently occupied by built-up development, active agricultural use, or environmentally sensitive designations^25,27^. In Beni Suef Governorate, such available land accounts for approximately 83.3% of the total governorate area, as illustrated in Fig. 2.

Land availability was incorporated into the AHP framework as a weighted criterion, reflecting its direct influence on construction feasibility, land-acquisition requirements, and long-term operational flexibility. Undeveloped barren land received the highest suitability scores, while built-up, agricultural, and sensitive land categories were assigned progressively lower values.

#### Geology criterion

Geological characteristics play a vital role in determining the suitability of sites for Waste-to-Energy (WTE) facilities by influencing construction feasibility and environmental stability. In Beni Suef Governorate, diverse geological formations are distributed across the area, each exhibiting varying degrees of stability. Geologically stable formations, including Eocene limestone and consolidated alluvial deposits, are preferred for (WTE) facility siting due to their superior bearing capacity and resistance to settlement (Fig. 3c). In contrast, unconsolidated deposits such as aeolian sand dunes and sabkha are considered least suitable owing to poor geotechnical properties and susceptibility to wind erosion and liquefaction.

#### Soil type

Soil characteristics significantly influence site suitability by affecting foundation stability, drainage capacity, and environmental impact. In Beni Suef Governorate, major soil types include Lithic Leptosol, Eutric Leptosol, Calcaric Fluvisol, and Calcaric Arenosol. Leptosols are typically shallow and rocky, presenting challenges for construction activities, while Fluvisols, commonly found near water bodies, are susceptible to flooding and erosion. In contrast, Arenosols and Calcisols exhibit better drainage and mechanical stability, making them more favorable for infrastructure development. Consequently, higher suitability weights are assigned to areas dominated by Arenosols and Calcisols. The spatial distribution of soil types is depicted in Fig. 3-d.

#### Distance to faults

Proximity to geological faults is a critical factor in site selection, given the potential risks of seismic activity and ground instability. Areas near active or potentially active faults are generally considered less suitable for infrastructure development due to increased hazards, such as earthquakes and ground displacement. The total length of faults in Beni Suef Governorate is approximately 223.3 km. Therefore, sites located farther from known faults are assigned higher suitability weights to minimize these risks. The distribution of faults and their distances from candidate sites were analyzed using geological maps and remote sensing data, as illustrated in Fig. 3-c.

All methodological procedures, including geospatial data acquisition, processing, and the administration of the expert consultation questionnaire for multi-criteria decision-making, were carried out in accordance with relevant academic and professional research guidelines.

### Limitations of standards

Determining the permissible buffer distances around Waste-to-Energy (WTE) facility sites requires careful consideration of governmental regulations, potential environmental hazards, public health concerns, and economic evaluations specific to each criterion. These restrictive criteria and recommended buffer values for Beni Suef Governorate are summarized in Table 3^23,24,30–33^ Following an extensive literature review and expert consultations, evaluation scores were assigned on a scale from 0 to 10, where 0 represents areas excluded from consideration, 1 indicates the least preferred zones, and 10 denotes the most suitable locations^12,24^. This scoring system was implemented in ArcGIS Pro to assess suitability levels at low and medium levels before integrating them into a multi-criteria decision-making (MCDM) framework alongside other factors. Consequently, highly acceptable locations for each criterion, along with their respective buffer zones and suitability ratings, were identified to guide optimal site selection in the governorate.

**Table 3:** Details and grade values for the ultimate criteria.

Criteria	Restricted	1	2	3	4	5	6	7	8	9	10
**Distance to sensitive land uses**	< 500 m	> 500 m	200-meter intervals spaced equally								> 2300
**Distance from agricultural land**	< 500 m	500–700	200-meter intervals spaced equally								> 2300
**Distance to surface water**	< 1000 m	> 1000	100-meter intervals spaced equally								> 1900
**Distance to landfills**	> 25,000	25,000–22,500	Spans of 2500 m spaced evenly								< 2500
**Distance to the electricity network**	> 200 m	> 9000	Spans of 1000 m spaced evenly								1000–200
**Distance to the road network**	< 300 m	> 2700	Spans of 300 m spaced evenly								> 300
**Distance to protected areas**	< 500 m	500–1000	500-meter intervals spaced equally								> 5000
**Population density (Per/100m)**	> 650	650 − 585	65-person intervals spaced equally								
**Distance to Railway Line**	< 300 m	> 300	100-meter intervals spaced equally								> 100
**Wind**	3.5–3.4	3.4–3.2	0.2 m/s intervals spaced equally								1.9–1.7
**Elevation**	> 360	360	Spans of 40 m spaced evenly								−40–40
**Slope**	> 45	> 30–45	5-degree intervals spaced equally								< 5
**Land availability**	Inhabited land										Abandoned land
**Geology**	Nile deposits	Alluvial deposits	Sand dunes- Quaternary deposits- Pliocene -Oligocene								Eocene
**Soil**	Calcic Fluvisol	Haplic Gypsisol	Protic Arenosol - Calcaric Arenosol -Calcaric Regosol -Eutric Regosol- Haplic Calcisol-Eutric Leptosol								Lithic Leptosol
**Distance to Faults**	< 500 m	500–1000	500-meter intervals spaced equally								> 5000

### Data preparation

The study relied on a diverse set of high-quality spatial datasets covering sensitive land uses, built-up areas, agricultural lands, surface water bodies, available land parcels, road and railway networks, electricity infrastructure, population density, elevation, slope, geology, soil types, and fault lines. All data sources are summarized in Table 5. Landsat-9 imagery was processed and classified using the Support Vector Machine (SVM) algorithm to extract land-use, agricultural, and water-body layers. Elevation and slope were derived from the ASTER GDEM at 30 m resolution. Road, railway, and electricity network data were sourced from OpenStreetMap, while landfill locations and waste-management records for Beni Suef were obtained from official governmental datasets. To assess classification reliability, an independent accuracy assessment was conducted using 300 stratified validation points distributed equally across the four land-cover classes (Table 4). Reference labels were assigned through visual interpretation of high-resolution imagery supported by ancillary spatial data. The confusion matrix returned an Overall Accuracy of 92.0% and a Kappa Coefficient of 0.893, confirming that the derived land-cover layers are sufficiently reliable for regional-scale GIS-MCDM suitability modelling.

**Table 4:** Confusion matrix and accuracy assessment of the SVM-based land-cover classification.

Classified/Reference	Agricultural lands	Barren land	Built-up land	Water bodies	Row total	User’s accuracy %
Agricultural lands	70	4	1	0	75	93.3
Barren land	5	67	3	0	75	89.3
Built-up land	2	6	66	1	75	88.0
Water bodies	0	1	1	73	75	97.3
Column total	77	78	71	74	300	
Producer’s accuracy %	90.9	85.9	93.0	98.6		

Water bodies achieved the highest classification performance, with a User’s Accuracy of 97.3% and a Producer’s Accuracy of 98.6%, reflecting the strong spectral contrast between open water and surrounding land cover in Landsat imagery. Agricultural lands also performed robustly, with a User’s Accuracy of 93.3% and a Producer’s Accuracy of 90.9%. Barren and built-up land returned comparatively lower values; 89.3% and 88.0% User’s Accuracy respectively. owing to spectral similarity between dry exposed surfaces and urban construction materials, a well-documented limitation in arid environments. Despite this, the overall accuracy and Kappa coefficient remain well within acceptable thresholds for the land-cover-based criteria used in this study.

All datasets were projected, standardized, and pre-processed within ArcGIS Pro. To ensure spatial consistency, all input layers were projected to a unified coordinate reference system and converted to a common raster analysis grid prior to reclassification and weighted overlay. The final cell size was harmonized to the finest reliable spatial resolution of the core raster datasets, while vector-based layers were converted to Euclidean distance rasters and resampled to the same grid. Euclidean distance provides a consistent and comparable proximity metric across all criteria; however, it does not account for road hierarchy, traffic conditions, or vehicle-routing constraints, and results relating to transport accessibility should therefore be treated as preliminary spatial indicators rather than quantified transport-cost estimates. Table 5 documents the full spatial inventory, reporting for each dataset the data type, resolution or map scale, acquisition or production date, source authority, and preprocessing procedure. For geological and soil datasets, the original map scale and publication year are noted given that these layers represent generalized regional constraints rather than site-specific engineering inputs. Suitability classes and buffer zones were subsequently reclassified to a unified 0–10 scale to form the basis for the weighted overlay analysis.

**Table 5:** Data collection sources.

Layer	Data type	Spatial resolution/scale	Date	Source authority	Preprocessing
Land use/land cover	Raster	Landsat-9, 30 m	July 2025	USGS Earth Explorer	SVM classification, accuracy assessment, reclassification
Agricultural land	Raster	30 m	July 2025	USGS Earth Explorer	Extracted from SVM LULC
Surface water	Raster	30 m	July 2025	USGS Earth Explorer	Extracted from SVM LULC
Landfills	Point/vector	Official locations	2025	Environmental Affairs Agency Beni Suef Governorate	Euclidean distance raster
Roads	Vector	OSM-derived	2025	BBBike/OSM	Euclidean distance raster
Electricity network	Vector	OSM-derived or official grid data	2025	BBBike/relevant authority	Euclidean distance raster
Population density	Raster	100 m	2025	World Pop	Resampled to common grid
Elevation	Raster	30 m	ASTER GDEM	ASTER GDEM	DEM preprocessing
Slope	Raster	30 m	ASTER GDEM	ASTER GDEM	Derived from DEM
Geology	Vector/map	1:500,000	1987	EGPC/Conoco	Digitization/reclassification
Soil	Vector/map	map scale if available	1975	Soil Association Map of Egypt/EUDASM	Digitization/reclassification
Faults	Vector	1:500,000 source map	1987	EGPC/Conoco	Euclidean distance raster
Wind	Raster/table	ERA5-Land resolution or station-based	2022	ERA5-Land/EMA	Wind speed processing
Protected areas	Vector	Topographic maps, OpenStreetMap, and ArcGIS basemap imagery were used only for spatial cross-checking and visual verification of boundary location.	2025	Topographic maps/OSM	Euclidean distance raster

### MCDA approach using the AHP method

**Fig. 4:**
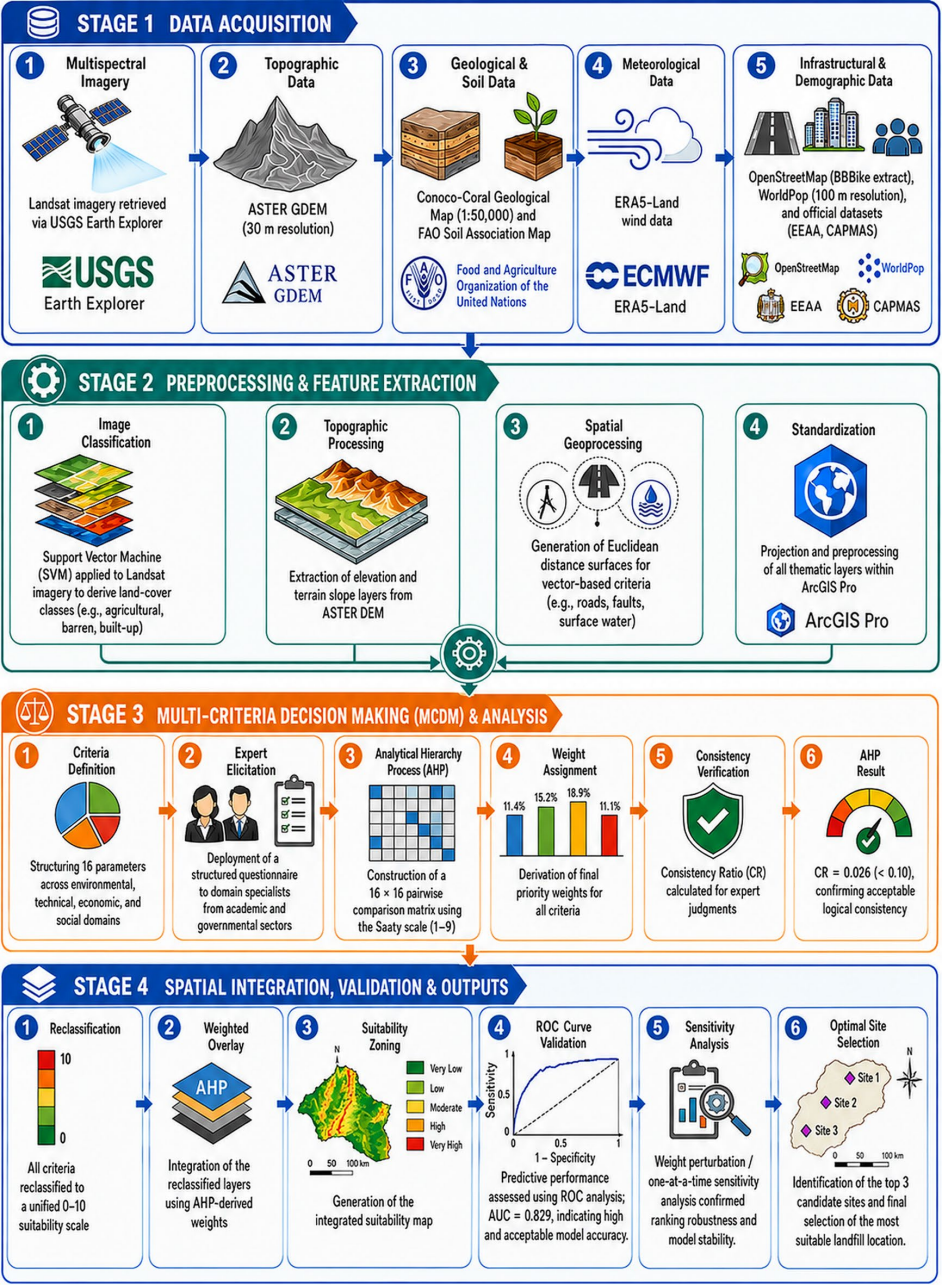
Flowchart of the adapted methodology used in this study.

**Fig. 5:**
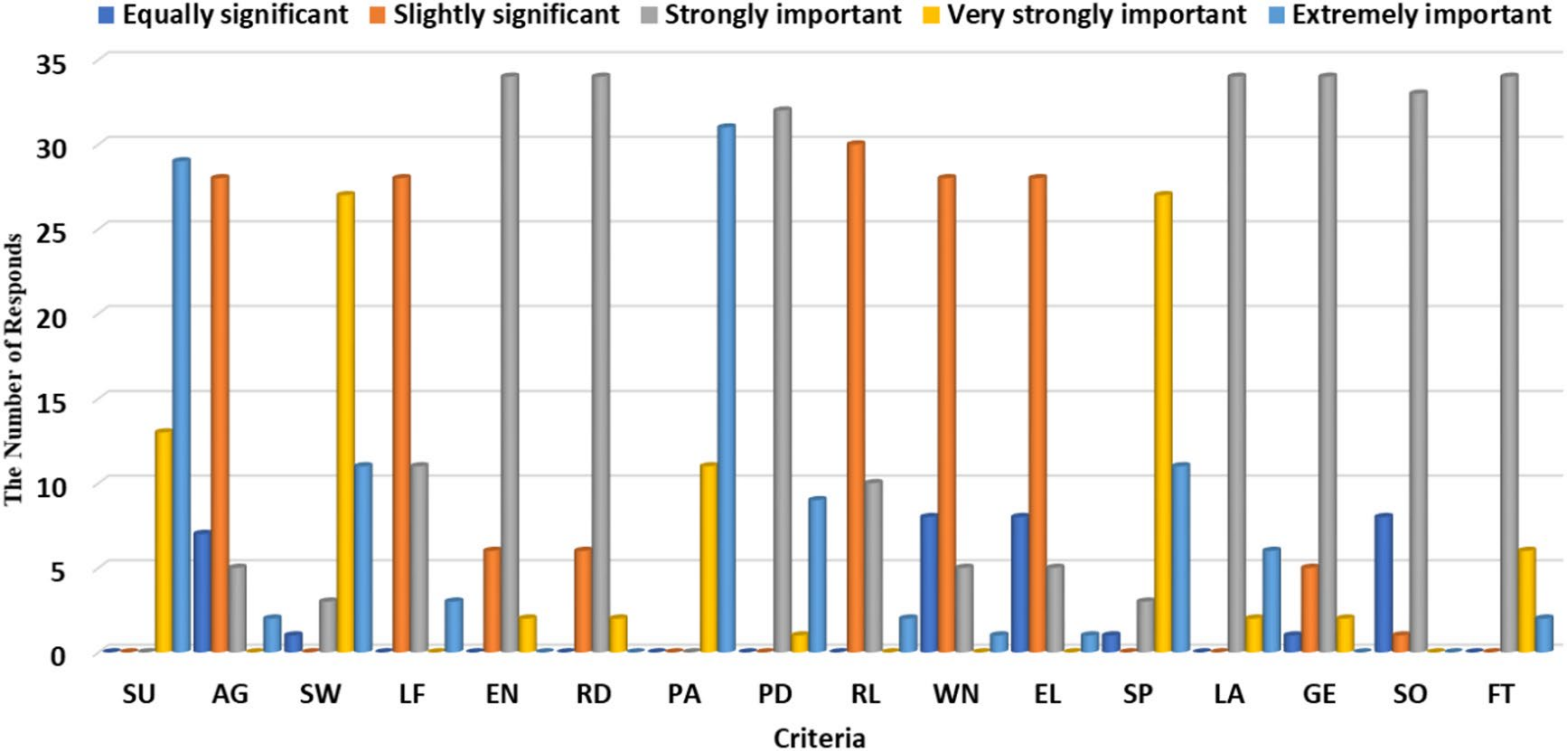
Questionnaire responses) https://forms.gle/2TjMiCQuPrKGD8RC9(: SU = Sensitive land; AG = Agricultural land; SW = Surface water; LF = Landfills; EN = Electricity network; RD = Road network; PA = Protected areas; PD = Population density; RL = Railway line; WN = Wind; EL = Elevation; SP = Terrain slope; LA = Land availability; GE = Geology; SO = Soil; FT = Faults.

In this study, the Analytic Hierarchy Process (AHP) method was applied to identify the most suitable site for establishing the facility in Beni Suef Governorate Fig. 4. To enhance the accuracy and reliability of the results, an electronic questionnaire was designed to assess the relative importance of the selection criteria based on expert judgments. A total of 42 experts from various relevant sectors participated, including representatives from the Ministry of Environment, the Solid Waste Management Department of the governorate, the Urban Planning Department, the Housing Directorate, the Ministry of Electricity, and academic institutions. The collected data were compiled and analyzed as presented in Table 6; Fig. 5.

**Table 6:** **Questionnaire responses)** https://forms.gle/2TjMiCQuPrKGD8RC9(.

Importance measure		Equally significant	Slightly significant	Strongly important	Very strongly important	Extremely important	Total	The weighted average of the responses on the significance value	Correction
Values AHP		1	3	5	7	9			
The Number of Responds	**SU**	0	0	0	13	29	42	8.38	8.5
	**AG**	7	28	5	0	2	42	3.19	3.0
	**SW**	1	0	3	27	11	42	7.24	7.0
	**LF**	0	28	11	0	3	42	3.95	4.0
	**EN**	0	6	34	2	0	42	4.81	5.0
	**RD**	0	6	34	2	0	42	4.81	5.0
	**PA**	0	0	0	11	31	42	8.48	8.5
	**PD**	0	0	32	1	9	42	5.90	6.0
	**RL**	0	30	10	0	2	42	3.76	4.0
	**WN**	8	28	5	0	1	42	3.00	3.0
	**EL**	8	28	5	0	1	42	3.00	3.0
	**SP**	1	0	3	27	11	42	7.24	7.0
	**LA**	0	0	34	2	6	42	5.67	5.5
	**GE**	1	5	34	2	0	42	4.76	5.0
	**SO**	8	1	33	0	0	42	4.19	4.0
	**FT**	0	0	34	6	2	42	5.48	5.5

#### Analytical hierarchical process (AHP)

The Analytic Hierarchy Process (AHP) is a multi-criteria decision-making technique based on an n × n pairwise comparison matrix, denoted A, that represents the relative importance of the evaluation criteria^34^. The application of this method begins with a clear definition of the research problem, followed by a detailed analysis of the relevant criteria and variables. Subsequently, the available alternatives are prioritized by constructing pairwise comparison matrices, and the process concludes with sensitivity or feasibility analyses to assess the robustness of the results^16^.

To compare the criteria listed in Table 6, the fundamental Saaty scale, ranging from 1 to 9, is used. A value of 1 indicates equal importance between two criteria, while a value of 9 reflects the extreme importance of one criterion over another^35^. The comparison values are assigned to the upper triangular part of the matrix, whereas the corresponding values in the lower triangular part are automatically determined using the reciprocity property. The general structure of the pairwise comparison matrix A is expressed as follows:

1 $$\:{\rm M}trix\:A\:=\:\left[\begin{array}{ccccc}1&\:{A}_{12}&\:{A}_{13}&\:\cdots\:&\:{A}_{1n}\\\:\frac{1}{{A}_{12}}&\:1&\:{A}_{23}&\:\cdots\:&\:{A}_{2n}\\\:\frac{1}{{A}_{13}}&\:\frac{1}{{A}_{23}}&\:1&\:\cdots\:&\:{A}_{3n}\\\:\vdots&\:\vdots&\:\vdots&\:\ddots\:&\:\vdots\\\:\frac{1}{{A}_{1n}}&\:\frac{1}{{A}_{2n}}&\:\frac{1}{{A}_{3n}}&\:\cdots\:&\:1\end{array}\right]$$

**Table 7:** AHP scale by Saaty.

Amount of Importance	Definition	Description
1	Equally significant compared to other things	Two factors that equally contribute to the goal
3	A little more significant than others	One criterion is moderately supported by the assessment more so than the other
5	Significantly more significant than others	In actuality, evaluation prefers one criterion over the other
7	Really significantly more significant than others	In comparison, the evaluation substantially favors one criterion over the other
9	Significantly more significant than others	The most valid evidence favors one criterion above another
2, 4, 6, 8	Value separating neighboring numbers	When to make a compromise
Opposite	Value for the comparison to the opposite	If one of the aforementioned integers exists between criterion *i* and criterion *j*, then *j* has a different value than *i*

Table 7 illustrates the Saaty importance scale adopted in this study. A value of 1 denotes equal contribution of two criteria to the objective, while values of 3, 5, 7, and 9 represent increasing degrees of preference, ranging from slight to extreme importance. Intermediate values (2, 4, 6, and 8) are used to express compromise judgments between adjacent importance levels. Reciprocal values are assigned for inverse comparisons.

Using matrix A, a normalized matrix N is generated according to Eq. (2) by dividing each element by the sum of its corresponding column. The priority weight vector W is then obtained by averaging each row of the normalized matrix. Evaluating the consistency of expert judgments is a fundamental step in the AHP methodology. This is achieved by calculating the Consistency Ratio (CR)^36^. A pairwise comparison matrix is considered acceptable when the CR value is less than 0.10 (10%). If this threshold is exceeded, the comparison matrix should be revised.

2 $$\:Matrix\:N\:=\:\left[\frac{{A}_{ij}}{{\varSigma\:}_{k=1}^{n}\:{A}_{kj}}\right]\:\:\:\:Vectored\:Pesos\:W$$

3 $$\:CR\:=\:\frac{CI}{RI}$$

4 $$\:CI\:=\:\frac{{\lambda\:}_{max}\:-\:n}{n\:-\:1}$$

5 $$\:{\lambda\:}_{max}\:=\:\left(A\right)\:\times\:\:\left(W\right)$$

It can also be calculated RI value from Table 8.

The Random Index (RI) values corresponding to each matrix dimension n were taken from Saaty’s^9^ standard tabulated values (Table 8), against which the Consistency Index (CI) was normalized to compute the Consistency Ratio (CR = CI/RI).

**Table 8:** The used Random Index (RI) values.

*N*	1	2	3	4	5	6	7	8	9	10
RI	0	0	0.58	0.90	1.12	1.24	1.32	1.41	1.45	1.49

### Model validation protocol

Model validation was conducted using Receiver Operating Characteristic (ROC) analysis to evaluate the ability of the final GIS-MCDM suitability model to discriminate between observed suitable and unsuitable validation points^37^. A total of 430 independent validation points were used, comprising 223 observed suitable points and 207 observed unsuitable points. The observed binary class of each point was compared with the predicted suitability score extracted from the final suitability raster. The Area Under the Curve (AUC) was calculated as a threshold-independent measure of overall model discrimination performance. The optimal suitability threshold was identified by maximizing the sum of sensitivity and specificity, and at this threshold a confusion matrix was constructed from which sensitivity, specificity, precision, overall accuracy, and Kappa coefficient were derived.

## Results and Discussion

### Analysis of the appropriateness of each criterion

**Fig. 6:**
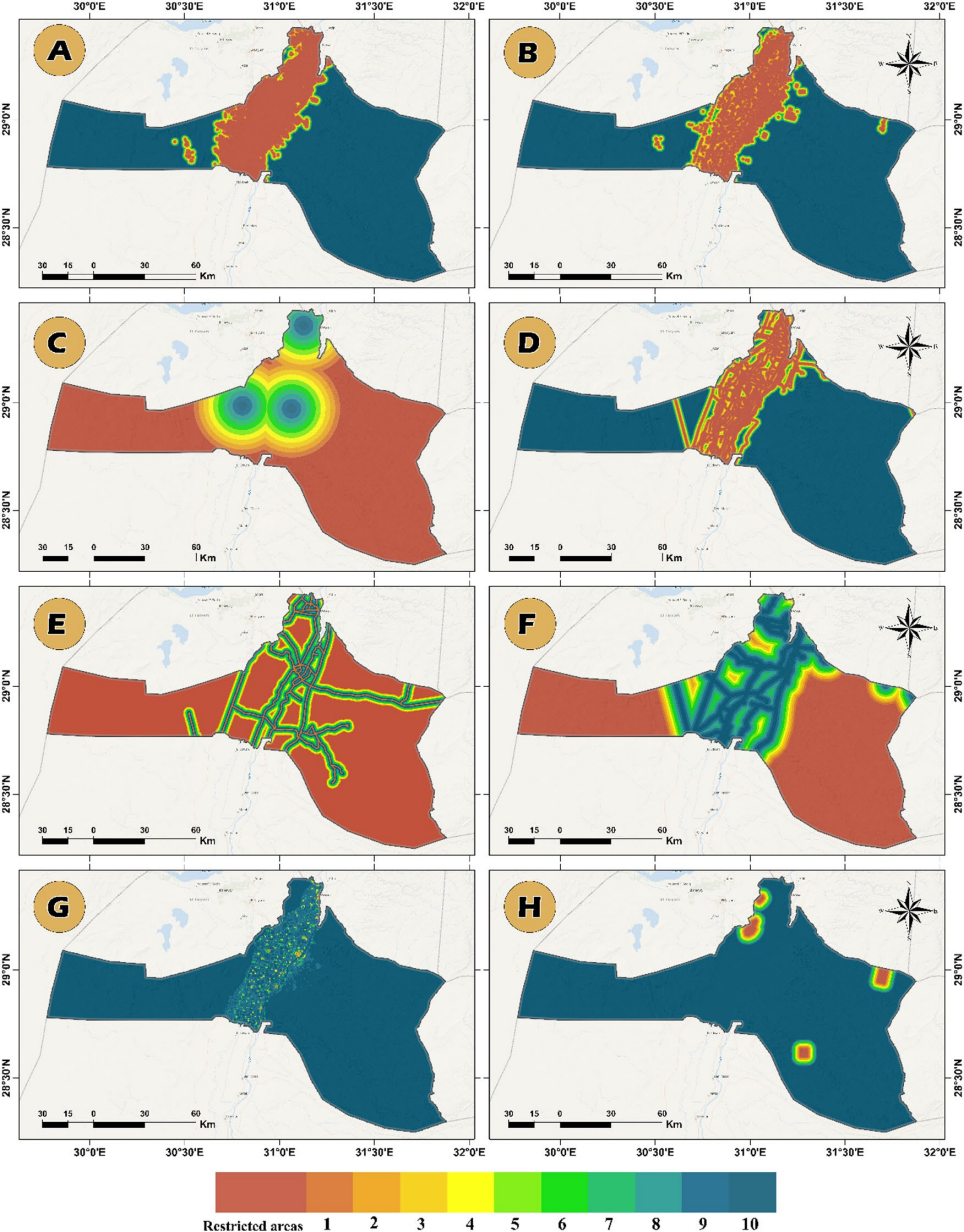
Displays the appropriateness rankings for each criterion in the following order: red (0) indicates a restricted region, 1–4 indicates low suitability, 5–7 indicates moderate suitability, and 8–10 indicates high suitability. A – Distance from agricultural land B – Distance to sensitive land uses C – Distance to landfills D – Distance to surface water E – Distance to the road network F – Distance to the electricity G – Population density H – Distance to protected area. This figure was created by ArcGIS Pro version 3.6. https://www.esri.com/en-us/arcgis/products/arcgis-pro/resources.

**Fig. 7:**
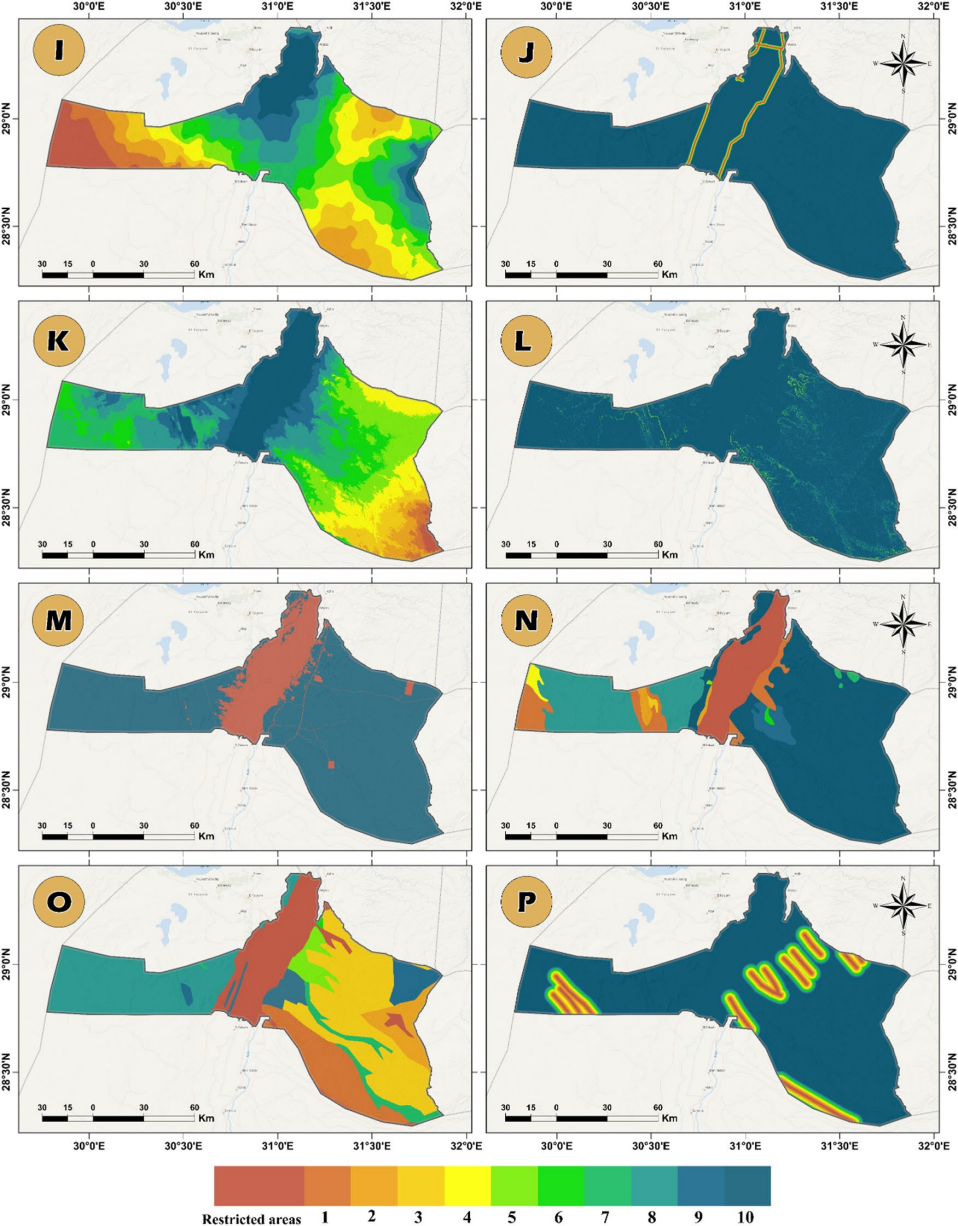
Displays the appropriateness rankings for each criterion in the following order: red (0) indicates a restricted region, 1–4 indicates low suitability, 5–7 indicates moderate suitability, and 8–10 indicates high suitability. I – Wind Speed J – Distance to Railway Line K – Elevation L – Terrain slope M – Land availability N – Geology O – Soil P – Distance to Faults. This figure was created by ArcGIS Pro version 3.6. https://www.esri.com/en-us/arcgis/products/arcgis-pro/resources.

Figures 6 and 7 show the outcomes of each criterion’s classification (0–10) using ArcGIS Pro^12,24^. The spatial classification of the suitability criteria for establishing a Waste-to-Energy (WTE) facility in Beni Suef Governorate (10,698.5 km²) reveals clear spatial differentiation across all investigated factors. The restricted zones (class 0) and highly suitable zones (class 10) vary significantly in terms of environmental sensitivity, infrastructure distribution, and geomorphological characteristics.

For the distance to sensitive land uses criterion, restricted zones occupy 1,570.8 km² (14.7%), reflecting the high concentration of urban centers, villages, and environmentally vulnerable receptors. In contrast, the highly suitable zones (class 10) account for 8,197.7 km² (76.6%), forming the broadest continuous areas available for potential facility placement. A similar pattern appears in the distance from agricultural land criterion, where restricted agricultural zones cover 1,983.6 km² (18.5%), whereas highly suitable non-agricultural areas cover 8,173.8 km² (76.4%), underscoring the dominance of barren and semi-desert regions.

Hydrological constraints are less dominant. The distance to surface water shows restricted areas totaling 1,400.2 km² (13.1%), while safe buffer zones (class 10) cover 8,271.2 km² (77.3%). In contrast, distance to landfills produces the greatest restricted extent, reaching 5,914.4 km² (55.3%), due to the clustering of uncontrolled disposal sites and their extended impact buffers. Only 189.6 km² (1.7%) fall within the highly suitable class.

Infrastructure-related criteria exhibit a more balanced distribution. For distance to the electricity network, restricted areas account for 6,563.1 km² (61.3%) and highly suitable zones account for 1,085.7 km² (10.1%). For distance to the road network, restricted zones cover 401.7 km² (3.8%), and highly suitable zones reach 7,772.5 km² (72.6%). A similar trend is observed for distance to the railway line, where restricted buffers cover only 94.7 km² (0.9%), while the highly suitable class covers 10,323.9 km² (96.5%).

Environmental protection criteria also reveal limited constraints. Distance to protected areas shows restricted zones of 132.6 km² (1.2%), whereas the highly suitable class reaches 9,930.1 km² (92.8%).

Population density also shows minimal restriction, with only 0.2 km² (< 0.01%) in class 0, while the highly suitable, low-density zones cover 9,523.9 km² (89%), reflecting the predominantly rural nature of the governorate.

Meteorological and topographic criteria also support suitability. Wind speed–based restricted zones cover 737.4 km² (6.9%), while highly suitable zones represent 891.5 km² (8.3%). Elevation produces 170.0 km² (1.6%) restricted and 105.4 km² (1%) highly suitable. Slope constraints are minimal, with only 0.3 km² (0.003%) restricted and a dominant 9,173.7 km² (85.8%) within the highly suitable class, confirming the governorate’s flat geomorphology.

Land availability shows an opposite pattern: restricted zones reach 1,914.0 km² (17.9%), while highly available land dominates with 8,784.5 km² (82.1%). Geology-related constraints are moderate: restricted zones comprise 1,353.4 km² (12.6%), whereas stable geological formations suitable for construction represent 6,078.6 km² (56.8%) in the highly suitable class. Similarly, soil constraints extend over 1,898.4 km² (17.7%), while optimal soil conditions occupy 328.4 km² (3.1%) in the class 10 category.

Finally, the distance-to-fault criterion reveals restricted seismic buffers totaling 223.5 km² (2.1%), while tectonically safe regions occupy 8,182.7 km² (76.5%), confirming the structural stability of most of Beni Suef.

Overall, combining all criteria indicates that restricted areas across the governorate range between 2 and 20% depending on the criterion, while highly suitable areas frequently exceed 70%, particularly in geology, slope, land availability, distance from settlements, infrastructure buffers, and seismic stability. These results provide a robust foundation for applying MCDM and AI-based spatial optimization models to identify the most sustainable locations for (WTE) facility development in Beni Suef Governorate.

### Interpretation of AHP-derived criterion weights

The expert questionnaire produced an aggregated importance score for each criterion using the 1–9 Saaty scale. The weighted average response for each criterion was first calculated and then adjusted to the nearest appropriate Saaty-scale value to reduce excessive decimal precision. These corrected scores were subsequently used to guide the construction of the pairwise comparison matrix Table 9, which encompassed 16 criteria and yielded 120 pairwise comparisons. The final AHP weights were derived from the normalized principal eigenvector of the full matrix, meaning that each criterion’s weight reflects its comparative position against all other criteria rather than its individual score alone.

The analysis produced a maximum eigenvalue (λmax) of 16.615, demonstrating strong numerical convergence achieved in four iterations at a tolerance of Δ = 4.5 × 10⁻⁸. The Consistency Ratio (CR) was 0.026 (2.6%), well below the accepted threshold of 10%, confirming that the expert judgments were logically coherent and free of contradictory preferences. The derived weights can therefore be applied with confidence as a reliable quantitative representation of criteria priorities within the spatial context of Beni Suef Governorate.

The final suitability surface was produced using the Suitability Modeler workflow in ArcGIS Pro 3.6, in which all standardized criterion rasters were transformed to a common 0–10 scale and combined according to their normalized AHP weights. The weighted linear combination is expressed as:

SI = Σ(W_i_ × X_i_).

where W_i_ is the normalized weight and X_i_ is the standardized suitability score of criterion i. Restricted areas were handled through Boolean constraint logic:

Final SI = [Σ(W_i_ × X_i_)] × C.

where C = 0 for restricted cells and C = 1 for cells available for evaluation. The site’s proximity to the regional road network represents a potential logistical advantage in terms of reducing haulage distances between waste-generation zones, existing landfill infrastructure, and the proposed WTE facility; however, this should be interpreted as a spatial proxy for transport efficiency rather than a quantified transport-cost estimate.

**Table 9:** Pairwise comparison matrix AHP scale by Saaty.

Criteria	SU	AG	SW	LF	EN	RD	PA	PD	RL	WN	EL	SP	LA	GE	SO	FT
SU	1.00	4.00	1.00	3.00	2.00	2.00	1.00	1.00	4.00	5.00	5.00	1.00	2.00	2.00	2.00	2.00
AG	0.25	1.00	0.33	1.00	0.50	0.50	0.25	0.33	1.00	1.00	1.00	0.33	0.50	0.50	1.00	0.50
SW	1.00	3.00	1.00	2.00	2.00	2.00	1.00	1.00	3.00	4.00	4.00	1.00	1.00	2.00	2.00	1.00
LF	0.33	1.00	0.50	1.00	1.00	1.00	0.33	0.50	1.00	2.00	2.00	0.50	0.50	1.00	1.00	0.50
EN	0.50	2.00	0.50	1.00	1.00	1.00	0.50	0.50	2.00	3.00	3.00	0.50	1.00	1.00	1.00	1.00
RD	0.50	2.00	0.50	1.00	1.00	1.00	0.50	0.50	2.00	3.00	3.00	0.50	1.00	1.00	1.00	1.00
PA	1.00	4.00	1.00	3.00	2.00	2.00	1.00	1.00	4.00	5.00	5.00	1.00	2.00	2.00	3.00	2.00
PD	1.00	3.00	1.00	2.00	2.00	2.00	1.00	1.00	1.00	1.00	1.00	1.00	1.00	1.00	1.00	1.00
RL	0.25	1.00	0.33	1.00	0.50	0.50	0.25	1.00	1.00	1.00	1.00	0.33	0.50	0.50	1.00	2.00
WN	0.20	1.00	0.25	0.50	0.33	0.33	0.20	1.00	1.00	1.00	1.00	0.25	0.33	0.50	0.50	0.33
EL	0.20	1.00	0.25	0.50	0.33	0.33	0.20	1.00	1.00	1.00	1.00	0.25	0.33	0.50	0.50	0.33
SP	1.00	3.00	1.00	2.00	2.00	2.00	1.00	1.00	3.00	4.00	4.00	1.00	1.00	2.00	2.00	1.00
LA	0.50	2.00	1.00	2.00	1.00	1.00	0.50	1.00	2.00	3.00	3.00	1.00	1.00	1.00	2.00	1.00
GE	0.50	2.00	0.50	1.00	1.00	1.00	0.50	1.00	2.00	2.00	2.00	0.50	1.00	1.00	1.00	1.00
SO	0.50	1.00	0.50	1.00	1.00	1.00	0.33	1.00	1.00	2.00	2.00	0.50	0.50	1.00	1.00	0.50
FT	0.50	2.00	1.00	2.00	1.00	1.00	0.50	1.00	0.50	3.00	3.00	1.00	1.00	1.00	2.00	1.00

Number of comparisons = 120 - Consistency Ratio CR = 2.6% - Principal eigen value = 16.615: SU = Sensitive land; AG = Agricultural land; SW = Surface water; LF = Landfills; EN = Electricity network; RD = Road network; PA = Protected areas; PD = Population density; RL = Railway line; WN = Wind; EL = Elevation; SP = Terrain slope; LA = Land availability; GE = Geology; SO = Soil; FT = Faults.

**Table 10:** The proposed criteria weights using the AHP method that have been applied.

Criteria	Priority (%)	Rank
SU	11.2	2
AG	3	14
SW	9.4	3
LF	4.2	12
EN	5.6	8
RD	5.6	8
PA	11.4	1
PD	7.3	5
RL	3.9	13
WN	2.7	15
EL	2.7	15
SP	9.4	3
LA	6.9	6
GE	5.5	10
SO	4.6	11
FT	6.6	7

SU = Sensitive land; AG = Agricultural land; SW = Surface water; LF = Landfills; EN = Electricity network; RD = Road network; PA = Protected areas; PD = Population density; RL = Railway line; WN = Wind; EL = Elevation; SP = Terrain slope; LA = Land availability; GE = Geology; SO = Soil; FT = Faults.

**Table 11:** Comparative Analysis of AHP-Method Criteria Weights for Decision-Making.

Criteria	Min	Max	Result
SU	9.2	13.2	11.2
AG	2.4	3.6	3
SW	3.4	11	9.4
LF	4.3	5	4.2
EN	4.3	6.9	5.6
RD	4.4	6.9	5.6
PA	4.4	13.4	11.4
PD	1.3	10.2	7.3
RL	1.4	6.5	3.9
WN	1.4	4	2.7
EL	1.4	4	2.7
SP	7.8	11	9.4
LA	5.5	8.3	6.9
GE	4.5	6.5	5.5
SO	3.5	5.7	4.6
FT	4.7	8.5	6.6

SU = Sensitive land; AG = Agricultural land; SW = Surface water; LF = Landfills; EN = Electricity network; RD = Road network; PA = Protected areas; PD = Population density; RL = Railway line; WN = Wind; EL = Elevation; SP = Terrain slope; LA = Land availability; GE = Geology; SO = Soil; FT = Faults.

**Fig. 8:**
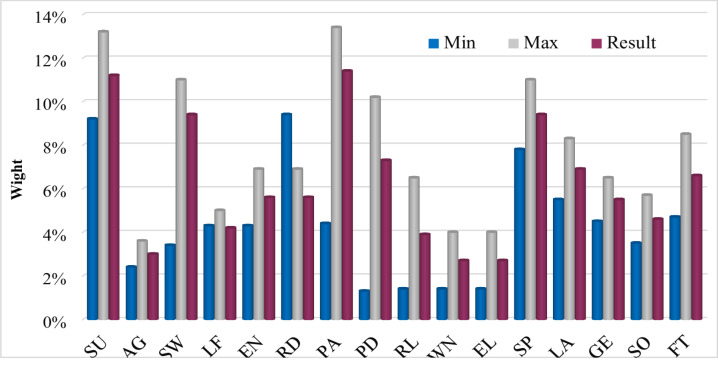
Comparative Analysis of AHP-Method Criteria Weights for Decision-Making.: SU = Sensitive land; AG = Agricultural land; SW = Surface water; LF = Landfills; EN = Electricity network; RD = Road network; PA = Protected areas; PD = Population density; RL = Railway line; WN = Wind; EL = Elevation; SP = Terrain slope; LA = Land availability; GE = Geology; SO = Soil; FT = Faults.

Regarding the interpretation of the derived weights, the results indicate that the experts assigned paramount priority to environmental and social criteria, treating them as governing prerequisites that supersede operational considerations. The criterion of Distance from Protected Areas ranked highest (PA = 11.4%), followed closely by Distance from Sensitive Land Uses (SU = 11.2%). Distance from Surface Water (SW = 9.4%) and Slope (SP = 9.4%) also fell within this high-importance tier. This hierarchy reflects expert consensus aimed at mitigating adverse impacts on natural resources and averting potential environmental and social pollution. Furthermore, the prominent weighting of the slope factor underscores its direct implications for structural safety, insulation, drainage, and stormwater management, critical practical considerations when siting an industrial facility of this magnitude Table 11 and Fig. 8.

The medium-importance tier exhibits a clear equilibrium among social acceptance, geological safety, and the project’s spatial configuration requirements. Population Density (PD = 7.3%) received significant weight, underscoring the experts’ intent to minimize community exposure to noise, odors, and truck traffic, thereby mitigating public opposition to waste management facilities. Concurrently, Land Availability (LA = 6.9%) emerged as a decisive operational element, ensuring adequate space for associated infrastructure (e.g., reception and sorting yards, storage facilities, safety zones, and traffic routing) while accommodating future expansion. Additionally, Distance from Faults (FT = 6.6%) reflects a geotechnical safety perspective aimed at reducing ground instability risks, which is vital for heavy-duty projects requiring robust foundations and sensitive operational equipment.

Supporting logistical and technical criteria obtained substantial weights, albeit lower than their environmental counterparts. This sequence logically mirrors standard spatial planning priorities. Proximity to the Electricity Network (EN = 5.6%) and Proximity to the Road Network (RD = 5.6%) held equal weight, aligning perfectly with the functional requirements of (WTE) plants: a reliable grid connection for electricity export and an efficient road network to ensure a sustainable waste supply while minimizing transportation time and costs. These were followed by Geology (GE = 5.5%) and Soil (SO = 4.6%), recognized as determinants for minimizing subsidence and seepage risks while enhancing foundation quality.

The remaining criteria included Distance from Landfills (LF = 4.2%), Proximity to Railways (RL = 3.9%), and Distance from Agricultural Land (AG = 3.0%). Finally, Wind Speed (WN = 2.7%) and Elevation (EL = 2.7%) recorded the lowest weights. This indicates that, within the specific context of Beni Suef, experts view these meteorological and topographical factors primarily as secondary optimization variables for differentiating between closely matched sites, rather than as fundamental, primary decision-making factors.

**Fig. 9:**
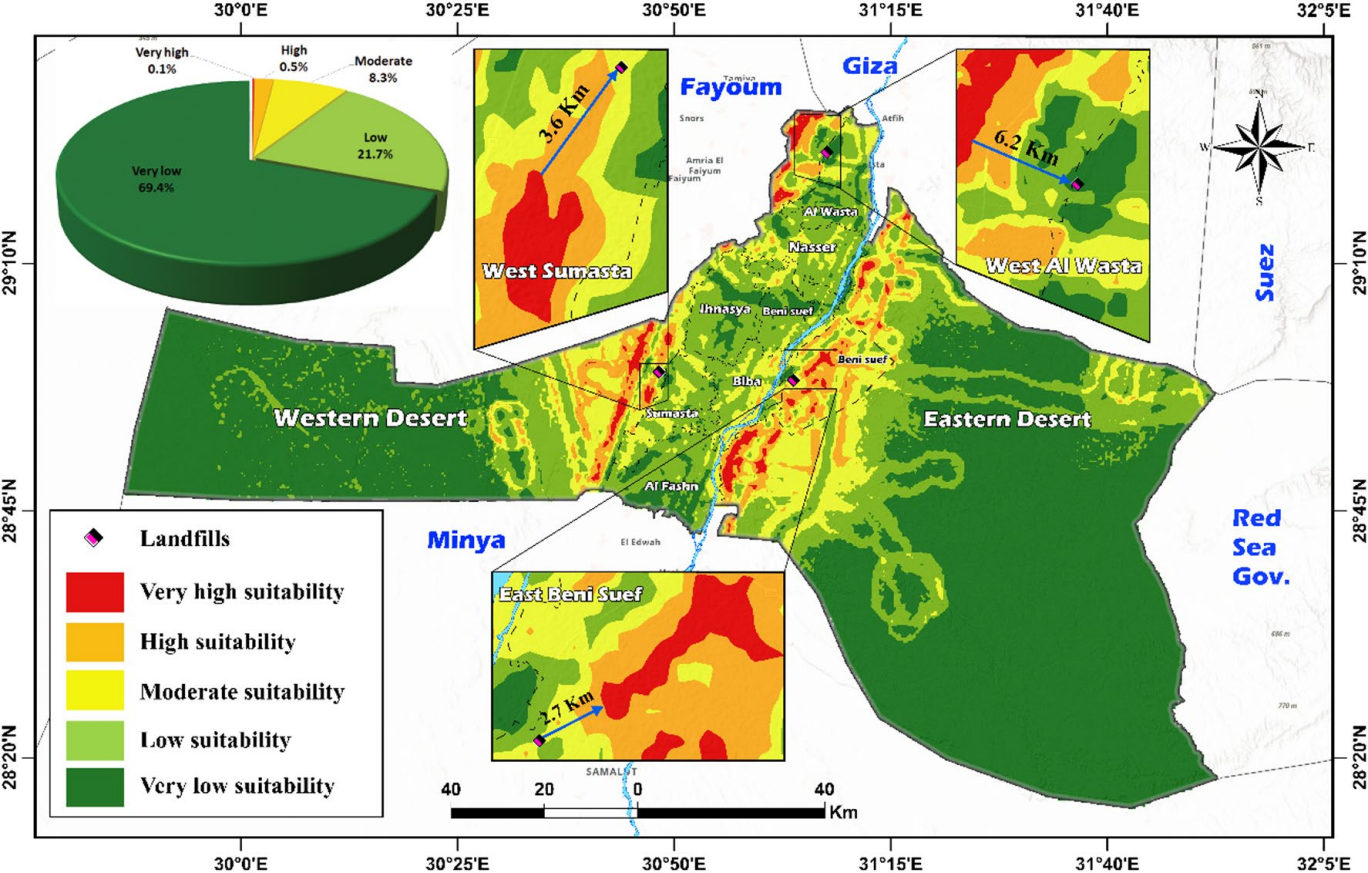
Integrated WTE plant site suitability map for Beni Suef Governorate, Egypt, derived using the GIS-MCDM/AHP framework. The map classifies the study area into five suitability levels — very high, high, moderate, low, and very low based on environmental, geological, hydrological, social, and infrastructural criteria. Map produced using ArcGIS Pro 3.6 (https://www.esri.com/en-us/arcgis/products/arcgis-pro/resources).

As illustrated in Fig. 9 and summarized in Table 12, the spatial distribution of suitability levels for waste-to-energy plant site selection in Beni Suef Governorate reveals a clear dominance of low and very low suitability classes. These two classes together account for approximately 90.8% of the total study area (9,749.1 km²), reflecting the strict environmental, infrastructural, and socio-economic constraints imposed in the AHP-based multi-criteria evaluation process. In contrast, areas classified as highly suitable and very highly suitable occupy only about 2.02% of the governorate’s total area (59.5 km²), indicating the limited availability of optimal locations that satisfy all selection criteria. Moderately suitable areas account for 7.2% of the total area, suggesting potential candidate sites that may be considered with additional technical or planning adjustments. These results highlight the effectiveness of the integrated GIS-AHP approach in narrowing down feasible locations and supporting evidence-based decision-making for sustainable waste-to-energy development in the governorate.

Furthermore, the spatial suitability analysis identified three candidate sites demonstrating high suitability scores (Fig. 9). The primary site, located in the eastern sector of the Beni Suef district, encompasses an area of 22.75 km². It is situated approximately 2.6 km from the nearest landfill, the Beni Suef landfill, which is the largest in the governorate. This proximity provides an optimal buffer distance for the logistical efficiency of disposing of residual ash and non-combustible waste post-operation. As illustrated in Fig. 9, the geometric center of this proposed site is located at coordinates 29°01′N, 31°07′E. This location emerges as the optimal alternative due to its strategic positioning and operational metrics. The adjacent landfill receives an average daily influx of 136.62 tons of waste. This volume represents approximately 26.2% of the total solid waste generated within the Beni Suef Governorate, thereby ensuring a critical and sustainable feedstock supply for the (WTE) plant. The site’s proximity to the regional road network suggests a potential logistical advantage by reducing likely haulage distances between waste-generation zones, existing landfill infrastructure, and the proposed WTE facility. However, this should be interpreted as a spatial proxy for transport efficiency rather than a quantified transport-cost estimate.

Based on a daily MSW input of 521 tons/day, equivalent to 521,000 kg/day, and assuming an indicative LHV of 6–10 MJ/kg^38^ and a conservative net electrical efficiency of 20–25%, the potential average net electricity output is estimated at approximately 7.2–15.1 MWe. At 8,000 operating hours per year, this corresponds to approximately 58–121 GWh/year. This estimate is intended as a planning-level indicator only; actual generation depends on waste composition, moisture content, measured LHV, selected (WTE) technology, plant availability, auxiliary power consumption, and emission-control requirements.

Environmental safety and community impact were also critical determinants in evaluating site suitability, with particular consideration given to wind dynamics. According to meteorological records from the Egyptian Meteorological Authority^39^, the prevailing winds in the study area are predominantly northwesterly. Given the selected site’s location in the governorate’s desert hinterland, this prevailing wind trajectory ensures that potential airborne emissions and odors are dispersed toward the deep, uninhabited desert. Consequently, this topographical and climatic configuration acts as a natural shield, effectively mitigating environmental risks and protecting both agricultural lands and populated urban centers from potential nuisances.

**Table 12:** Spatial distribution of land suitability classes and their corresponding areas.

Suitability level	Area Km^2^	%
Very high	6.3	0.19
High	53.2	1.83
Moderate	889.9	7.2
Low	2321.5	21.48
Very low	7427.6	69.3
Total	**10698.5**	**100**

The integrated suitability map identifies three candidate WTE zones within the highest suitability class (Fig. 9). Of these, Site A stands out as the primary candidate, owing to its position relative to existing landfill infrastructure, the regional road network, and the surrounding land-use pattern. Its location east of Beni Suef city, approximately 2.6 km from the governorate’s largest active landfill, offers both a reliable waste feedstock and logistical accessibility that the other two sites do not match to the same degree. Sites B and C nonetheless fall within the highest suitability class and should not be dismissed; both warrant the same level of field verification, environmental impact assessment, geotechnical investigation, and techno-economic review before any commitment to a final site is made.

It should be noted that this study was designed as a regional screening exercise, not a detailed engineering feasibility study. For this reason, fine-scale distance mapping and site-level proximity analysis were not pursued here. These are properly the work of the next stage, once a shortlist of sites has been agreed and resources can be directed toward site-specific investigation.

This study adopted a standard methodological framework for applying Multi-Criteria Decision Making (MCDM) techniques, utilizing ArcGIS Pro to facilitate the spatial site selection process. The evaluation criteria and their respective relative weights were established through a structured questionnaire administered to 42 domain experts. Their professional judgments were synthesized to calculate the criteria weights using the Analytic Hierarchy Process (AHP) (Table 10). During the pairwise comparison phase, the Consistency Ratio (CR) was calculated to validate the reliability of the expert judgments. Methodologically, a CR threshold of ≤ 0.1 (10%) is required to ensure logical consistency. In this study, the computed CR was 0.026 (2.6%), demonstrating a high degree of coherence and reliability in the evaluation outcomes.

These findings align with several existing studies. For instance, research conducted in the Izmir Metropolitan Municipality^10^ revealed that approximately 97% of the study area was geographically restricted and unsuitable for facility construction. In that context, proximity to transfer stations and the availability of municipal solid waste emerged as the most critical criteria, while elevation and slope ranked lowest. This hierarchy reflects the pivotal role of economic variables in minimizing operational expenditures. Similarly, a spatial assessment in Pathumthani^41^identified distance from urban areas, major water sources, and power plants as the paramount criteria, whereas proximity to railways and airports exerted the least influence. Furthermore^25^, found that distance from sensitive land uses and proximity to sanitary landfills recorded the highest weights, with surface slope and distance from airports deemed least important.

Collectively, this comparative analysis indicates that the relative importance of siting criteria is highly context-dependent, varying geographically according to the specific environmental, topographic, and economic characteristics of the study area. However, a broader review of the literature confirms a consistent trend: economic and environmental criteria predominantly secure the highest relative weights. This consensus underscores the critical objective of siting waste-to-energy (WTE) facilities in locations that simultaneously optimize operational cost-efficiency, preserve the surrounding ecosystem, and strictly mitigate any adverse externalities associated with such industrial infrastructure.

### Model validation and threshold-based sensitivity assessment

The predictive performance of the GIS-MCDM WTE plant site suitability model was evaluated using Receiver Operating Characteristic (ROC) analysis and threshold-based classification metrics^37^. A total of 430 independent validation points were used, comprising 223 observed suitable points and 207 observed unsuitable points. All records were retained, confirming that the validation dataset was complete and free of invalid or missing values (Table 13).

**Table 13:** Integrated validation and threshold-based sensitivity assessment of the GIS-MCDM WTE plant site suitability model.

Category	Indicator	Result
Validation sample	Total points	430
	Suitable/Unsuitable points	223/207
	Removed invalid records	0
ROC assessment	AUC	0.829
	Optimal threshold	2.603
	Threshold criterion	Max(Sensitivity + Specificity)
Confusion matrix	TP/FN	180/43
	TN/FP	207/0
Accuracy metrics	Sensitivity	0.807
	Specificity	1.000
	Precision	1.000
	Overall accuracy	0.900
	Kappa coefficient	0.801
Planning implication	Decision rule	Suitability score ≥ 2.603
	Screening behavior	Conservative (zero false positives)

Note: TP = true positives; TN = true negatives; FP = false positives; FN = false negatives. The optimal threshold was identified by maximizing the sum of sensitivity and specificity. The decision rule ≥ 2.603 defines the cut-off applied to classify predicted suitability scores into suitable and unsuitable classes for validation purposes.

**Fig. 10:**
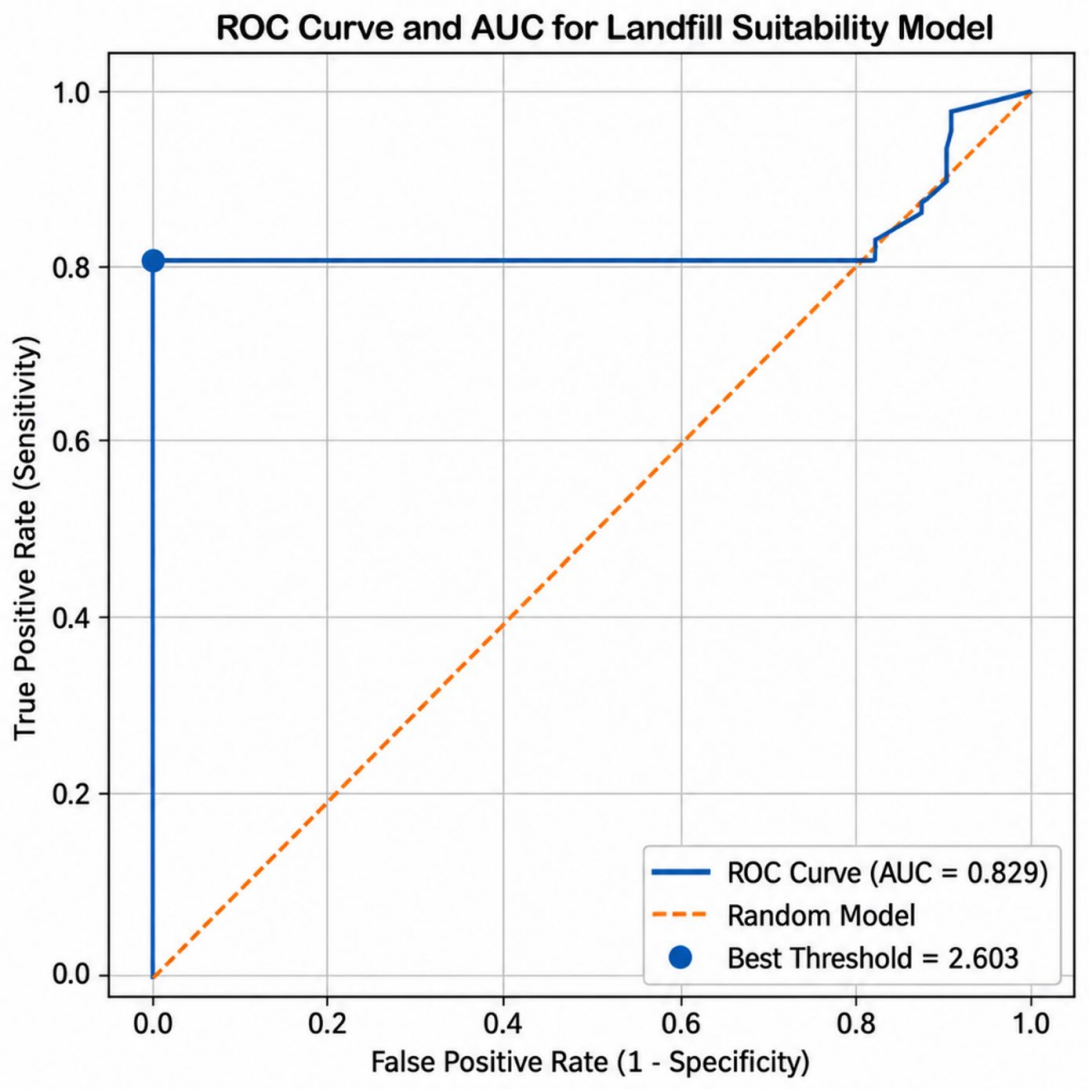
Receiver Operating Characteristic (ROC) curve and Area Under the Curve (AUC) for validating the GIS-MCDM WTE plant site suitability model. The curve was generated using 430 validation points comprising 223 observed suitable and 207 observed unsuitable locations. The model achieved an AUC of 0.829, indicating good discriminatory performance. The optimal threshold, identified by maximising the sum of sensitivity and specificity, was 2.603.

The ROC curve confirms that the suitability model performs substantially better than a random classifier. The AUC value of 0.829 reflects good discriminatory capacity between suitable and unsuitable locations, meaning that the model reliably assigns higher suitability scores to genuinely suitable sites than to unsuitable ones. Taken together, these results support the use of the GIS-MCDM framework as a reliable regional-scale screening tool for WTE facility site selection in Beni Suef Governorate Fig. 10.

At the optimal threshold of 2.603, the model achieved a sensitivity of 0.807 and a perfect specificity of 1.000. The sensitivity value indicates that 80.7% of observed suitable locations were correctly identified, while the specificity value confirms that every observed unsuitable location was correctly excluded. This performance pattern is particularly valuable for WTE facility siting, where falsely identifying unsuitable land as suitable can lead to environmental conflicts, engineering constraints, public opposition, or regulatory rejection.

The confusion matrix further illustrates the conservative and risk-averse character of the model. At the optimal threshold, 180 suitable points were correctly classified as suitable and 43 suitable points were classified as unsuitable, giving a false negative count of 43. Critically, all 207 observed unsuitable points were correctly classified, and no false positives were recorded. The complete absence of false positives is a meaningful planning advantage, as it reduces the likelihood of advancing genuinely unsuitable locations into costly feasibility, EIA, or engineering-investigation stages.

Overall accuracy reached 0.900, meaning that 90.0% of all 430 validation points were correctly classified at the optimal threshold. The Kappa coefficient of 0.801 indicates strong agreement between the observed validation classes and the predicted suitability classification beyond what would be expected by chance. The convergence of AUC, sensitivity, specificity, overall accuracy, and Kappa values provides consistent evidence that the model is robust and internally reliable for identifying priority WTE candidate sites at the regional planning scale.

From a planning perspective, the decision rule of suitability score ≥ 2.603 should be applied as a conservative threshold for prioritizing candidate WTE locations. Sites meeting this criterion are not ready-made construction sites; rather, they represent priority zones warranting detailed field verification, geotechnical investigation, environmental impact assessment, waste-transport modelling, and techno-economic feasibility analysis before any final siting commitment is made.

### Comparison of criterion weights with published GIS-AHP studies

The weight hierarchy derived in this study, with environmental protection and social proximity criteria (PA: 11.4%, SU: 11.2%, SW: 9.4%) dominating over logistical and geotechnical parameters, is broadly consistent with findings from comparable GIS-AHP (WTE) siting studies in international literature, while exhibiting regionally specific features that are discussed below.

Yaman^10^ conducted a GIS-AHP suitability analysis for (WTE) facility siting in the greater municipality of Izmir, Turkey, and reported that proximity to waste transfer stations and MSW generation density were the highest-weighted criteria, while elevation and slope ranked lowest. This contrasts with the present study, in which terrain slope (9.4%) is significantly higher, reflecting the more complex topography of the desert escarpment environment. Conversely, the dominance of transport proximity criteria in the Izmir study reflects a mature, geographically compact urban context in which the primary discriminating factor is operational efficiency rather than environmental exclusion, a distinction that underscores the importance of context-specific criterion selection in GIS-MCDM frameworks.

Chullamon and Skolpap^40^ applied GIS-AHP to (WTE) siting in Pathum Thani Province, Thailand, and found that distance from urban areas, proximity to water sources, and distance from power plant substations were the dominant criteria, with railways and airports ranked lowest. The high weight assigned to water source proximity in that study closely parallels the importance of surface water distance (SW: 9.4%) in our framework, consistent with (WTE) planners’ universal sensitivity to surface water contamination risks. The lower weight assigned to geological criteria in the Thai context is expected, given the comparatively lower seismic activity and more homogeneous alluvial geology of central Thailand relative to the fault-dissected arid terrain of Upper Egypt.

Abushammala et al^25^., working in Oman, an arid Gulf country with geographic similarities to the Egyptian Eastern Desert, reported that distance from sensitive land uses and distance from sanitary landfills received the highest weights, while slope and airport proximity received the lowest. The alignment between the sensitive land use weighting in the Omani study and the present study (SU: 11.2%, rank 2) suggests a consistent prioritization of social proximity constraints across Arabic-speaking, arid-region contexts. The absence of geological hazard criteria (faults, bearing capacity) in the Oman study represents a gap that the present work explicitly addresses, and the inclusion of fault proximity as a mid-tier criterion (FT: 6.6%) reflects the heightened geotechnical risk context of the Eastern Desert margin.

In Egypt, Elbeih et al^15^. conducted GIS-based landfill siting in Alexandria and reported that hydrogeological criteria, proximity to population, and road accessibility were key factors. The weight structure in the Alexandria study emphasized proximity to infrastructure, reflecting the dense urban geography of the coastal Delta context, in contrast to the present study’s emphasis on environmental exclusion criteria, a difference consistent with the fundamentally different planning environment of Upper Egypt’s arid interior. Similarly, the Nile Delta studies reviewed by Armanous et al^41^. consistently placed greater emphasis on avoiding agricultural land, reflecting the higher proportion of agricultural land in the Delta compared with the 12.2% agricultural coverage in Beni Suef.

### Limitations and future research directions

Like any regional-scale planning study, this work has a number of limitations that should be kept in mind when interpreting the results. The geological data used — the EGPC–Conoco map at 1:500,000 scale — was appropriate for the spatial screening carried out here, but it is not detailed enough to support final engineering decisions. The three shortlisted sites will each need dedicated geotechnical investigation, covering boreholes, in-situ testing, laboratory analysis, and foundation assessment, before any construction commitment can responsibly be made.

The AHP weighting procedure, though grounded in consultation with 42 experts drawn from multiple professional sectors, is by nature a subjective exercise. The weights reflect a collective judgment made at a particular moment, and they could reasonably shift if regulatory priorities, technology costs, or environmental conditions change. The study also works with a static picture of waste generation; it does not account for how urbanization over the coming decades might alter the spatial distribution of MSW feedstock and, with it, the relative appeal of the candidate sites.

On the transport side, proximity to roads and landfills was measured using straight-line Euclidean distance rather than a routed network model. This means the study cannot provide estimates of actual travel time, haulage cost, or fuel consumption for each site. A proper network-based analysis one that accounts for road type, vehicle capacity, collection routes, and district-level waste flows would give a much clearer picture of the true logistical differences between the three candidates, and this is an obvious priority for follow-up work.

Although the AHP weights passed internal consistency checks, the study did not carry out a systematic sensitivity analysis to explore what would happen to the suitability map if the weights were varied. Applying Monte Carlo simulation or structured weight-perturbation scenarios, particularly for the geotechnical, environmental, and social criteria, would help establish how robust the high-suitability zones are under different expert-judgment assumptions.

Finally, the ROC–AUC validation confirms that the model discriminates well at the regional scale, but this should not be mistaken for a site-level endorsement. The analysis does not replace the Environmental Impact Assessment that Egyptian law requires before any WTE facility can proceed. Air dispersion modelling, odor assessment, ash and leachate management, groundwater vulnerability, traffic impacts, and cumulative health risk evaluation all remain necessary steps in the project development process.

Looking ahead, several directions stand out as particularly worthwhile. Sentinel-1 InSAR monitoring could provide early evidence of ground deformation or subsidence at the shortlisted sites. Deep learning classification of high-resolution imagery would sharpen the land-cover layers that underpin the suitability model. Extending the framework to neighboring Upper Egyptian governorates Minya, Assiut, and Sohag would help build a coherent regional picture of WTE siting potential. And an economic optimization model linking facility costs with district-level transport distances could identify the most cost-effective spatial allocation of facilities across the region.

## Conclusions

This study has presented the first comprehensive, sixteen-criterion GIS-AHP site suitability assessment for Waste-to-Energy facility development in Beni Suef Governorate, Upper Egypt. The key findings and contributions are summarized below.

Municipal solid waste generation in the governorate has grown from 455.8 tons/day in 2020 to 521.7 tons/day in 2025 and is projected to reach 695.7 tons/day by 2035 a 33.4% increase over a decade that already exceeds the capacity of existing waste management infrastructure. This trajectory represents a quantified environmental and public health case for WTE investment that is difficult to ignore.

The sixteen-criterion analytical framework, developed through structured consultation with 42 experts from academic, governmental, and engineering backgrounds, returned an AHP consistency ratio of 2.6%, confirming the internal coherence of the weight elicitation process. The weight hierarchy — led by distance to protected areas (11.4%), sensitive land uses (11.2%), surface water (9.4%), and terrain slope (9.4%) — reflects the expert panel’s clear prioritization of environmental protection over logistical efficiency, a pattern consistent with comparable GIS-AHP siting studies in MENA and Mediterranean contexts.

The integrated suitability map shows that genuinely suitable WTE land is scarce: high and very high suitability zones together cover only 2.02% of the study area (59.5 km²), with a further 7.2% classified as moderate. The dominance of very low suitability across 69.3% of the governorate reflects the cumulative effect of overlapping environmental exclusion constraints rather than any fundamental barrier to WTE development in the region.

Model performance was strong across all validation metrics. ROC–AUC analysis returned an AUC of 0.829, an overall accuracy of 90.0%, and a Kappa coefficient of 0.801. At the optimal threshold of 2.603, the model achieved perfect specificity with no false positives, demonstrating conservative and risk-averse screening behavior that is well suited to preliminary WTE site selection.

Three candidate sites were identified within the high-suitability zones. Site A covering 22.75 km² east of Beni Suef city center (29°01′N, 31°07′E) achieved the highest composite suitability score. Its proximity to the governorate’s principal landfill (~ 2.6 km), favorable wind orientation relative to populated areas, geologically competent limestone substrate, and manageable terrain slope collectively reduce both environmental risk and anticipated capital development cost.

A notable contribution of this work is the explicit incorporation of geological hazard criteria fault proximity, lithological stability, and soil suitability which together account for 16.7% of the analytical weight. The inclusion of these criteria improves the physical realism of the suitability model in the arid Eastern Desert margin, though future spatial sensitivity analysis is needed to quantify the extent to which alternative weighting scenarios may influence the distribution of high-suitability zones.

The methodology developed here constitutes a reproducible, science-based decision-support tool applicable to Egyptian environmental planning under EEAA oversight and the mandates of the SWMRA (Law 202/2020), and it directly supports Egypt’s Sustainable Development Strategy 2030 targets for renewable energy diversification and the reduction of open dumping.

For practitioners and decision-makers, the study recommends prioritizing EIA and engineering feasibility processes for Site A as the most viable near-term WTE development location, investing in road and electricity network extension into the eastern desert margin to unlock moderate-suitability zones before 2030, adopting the sixteen-criterion framework as a standard siting protocol for Upper Egyptian waste management facilities, and incorporating WTE into regional integrated solid waste management plans as a key component of Egypt’s circular economy transition.

From an economic-planning perspective, the candidate sites should be understood as locations with potentially favorable spatial conditions rather than economically verified alternatives. The GIS-MCDM model captures spatial indicators that influence economic performance proximity to roads, electricity networks, and existing landfill infrastructure but a full techno-economic feasibility study remains necessary to quantify capital costs, operating costs, waste-transport costs, grid-connection costs, ash-management costs, and potential electricity revenues before any investment decision is made.

## Data Availability

The data presented in this study are available on request from the corresponding author.

## Abbreviations

AHP: Analytic Hierarchy Process
CAPMAS: Central Agency for Public Mobilization and Statistics
CI: Consistency Index
CR: Consistency Ratio
DEM: Digital Elevation Model
EEAA: Egyptian Environmental Affairs Agency
EGPC: Egyptian General Petroleum Corporation
EIA: Environmental Impact Assessment
FiT: Feed-in Tariff
GIS: Geographic Information Systems
AUC: Area Under the Curve
MCDM: Multi-Criteria Decision Making
MSW: Municipal Solid Waste
NDC: Nationally Determined Contribution
RI: Random Consistency Index
SDS: Sustainable Development Strategy
SVM: Support Vector Machine
SWMRA: Solid Waste Management Regulatory Authority
UTM: Universal Transverse Mercator
WLC: Weighted Linear Combination
WTE: Waste-to-Energy
ROC: Receiver Operating Characteristic

## References

[1] Kaza, S., Yao, L. C., Bhada-Tata, P., Van Woerden, F. & Levine, D. *What a Waste 2.0: A Global Snapshot of Solid Waste Management to 2050* (World Bank, 2018). 10.1596/978-1-4648-1329-0

[2] Hoornweg, D. What a Waste: A Global Review of Solid Waste Management. (2012).

[3] Egyptian Ministry of Environment / EEAA. State of the Environment Report 2023. (2023).

[4] Central Agency for Public Mobilization and Statistics (CAPMAS). *Population and Housing Census Preliminary Results, Beni Suef Governorate*. (2024). https://www.capmas.gov.eg/

[5] Lombardi, L., Carnevale, E. & Corti, A. A review of technologies and performances of thermal treatment systems for energy recovery from waste. *Waste Manag*. **37**, 26–44 (2015). 25535103 10.1016/j.wasman.2014.11.010

[6] Malinauskaite, J. et al. Municipal solid waste management and waste-to-energy in the context of a circular economy and energy recycling in Europe. *Energy* **141**, 2013–2044 (2017).

[7] Demesouka, O. E., Vavatsikos, A. P., Anagnostopoulos, K. P. & Spatial, U. T. A. S-UTA) – A new approach for raster-based GIS multicriteria suitability analysis and its use in implementing natural systems for wastewater treatment. *J. Environ. Manage.* **125**, 41–54 (2013). 23644589 10.1016/j.jenvman.2013.03.035

[8] Levaggi, L., Levaggi, R., Marchiori, C. & Trecroci, C. Waste-to-Energy in the EU: The Effects of Plant Ownership, Waste Mobility, and Decentralization on Environmental Outcomes and Welfare. *Sustainability* 12, 5743 (2020).

[9] Saaty, T. L. How to make a decision: The analytic hierarchy process. *Eur. J. Oper. Res.* **48**, 9–26 (1990).

[10] Yaman, A. A GIS-based multi-criteria decision-making approach (GIS-MCDM) for determination of the most appropriate site selection of onshore wind farm in Adana, Turkey. *Clean. Technol. Environ. Policy*. **26**, 4231–4254 (2024).

[11] Pathak, S. G. I. S. Based Multi-Criteria Decision Analysis For Waste Disposal Site Selection: A Case Study of Surkhet. (2024).

[12] Hereher, M., Al-Awadhi, T. & Mansour, S. Assessment of the optimized sanitary landfill sites in Muscat, Oman. *Egypt J. Remote Sens. Space Sci* **23**, 355–362 (2019).

[13] Rezaeisabzevar, Y., Bazargan, A. & Zohourian, B. Landfill site selection using multi criteria decision making: Influential factors for comparing locations. *J. Environ. Sci.* **93**, 170–184 (2020). 10.1016/j.jes.2020.02.030 32446453

[14] Mallick, J. et al. Modeling Groundwater Potential Zone in a Semi-Arid Region of Aseer Using Fuzzy-AHP and Geoinformation Techniques. *Water* **11**, 2656 (2019).

[15] El-Badrawy, H.T., Abo Khashaba, S.M., Araffa, S.A.S. et al. Mapping and predicting groundwater accumulations using remote sensing and aeromagnetic data: a case study from Bahariya Oasis, Western Desert, Egypt. *Sci Rep* **16**, 10489 (2026). 41888188 10.1038/s41598-026-42907-z PMC13031932

[16] Basha, A., Salem, A., Mostafa, W. & Farhan, M. H. Application of GIS Models in Determining the Suitable Site for a Solid Waste to Energy Plant in an Urban Area. *Civ. Eng. J.* **10**, 171–188 (2024).

[17] Ibrahim, M. I. M. & Mohamed, N. A. E. M. Towards Sustainable Management of Solid Waste in Egypt. *Procedia Environ. Sci.* **34**, 336–347 (2016).

[18] Elghazouly, H. G., Elnaggar, A. M., Ayaad, S. M. & Nassar Eman. T. Framework for integrating multi-criteria decision analysis and geographic information system (MCDA-GIS) for improving slums interventions policies in Cairo, Egypt. *Alex Eng. J.* **86**, 277–288 (2024).

[19] Arab Republic of Egypt. - General Census for Population, Housing and Establishments. (2017). https://censusinfo.capmas.gov.eg/metadata-en-v4.2/index.php/catalog/621

[20] Yigezu, Y., Bishaw, Z., Moustafa, M. & Saleh, E. *Political Economy of Wheat Sector in Egypt … Political Economy of the Wheat Sector in Egypt See…)*. (2025).

[21] GOPP. (2025). https://gopp.gov.eg/

[22] Panepinto, D. & Zanetti, M. C. Municipal solid waste incineration plant: A multi-step approach to the evaluation of an energy-recovery configuration. *Waste Manag*. **73**, 332–341 (2018). 28774585 10.1016/j.wasman.2017.07.036

[23] Hu, H., Li, X., Nguyen, A. & Kavan, P. A. Critical Evaluation of Waste Incineration Plants in Wuhan (China) Based on Site Selection, Environmental Influence, Public Health and Public Participation. *Int. J. Environ. Res. Public. Health*. **12**, 7593–7614 (2015). 26184242 10.3390/ijerph120707593 PMC4515677

[24] Tavares, G., Zsigraiová, Z. & Semiao, V. Multi-criteria GIS-based siting of an incineration plant for municipal solid waste. *Waste Manag*. **31**, 1960–1972 (2011). 21600754 10.1016/j.wasman.2011.04.013

[25] Abushammala, M. F. M., Qazi, W. A., Frrag, S., Alazaiza, M. Y. D. & Younes, M. K. Site selection of municipal solid waste incineration plant using GIS and multicriteria decision analysis. *J. Air Waste Manag Assoc.* **72**, 1027–1039 (2022). 35404762 10.1080/10962247.2022.2064002

[26] Egyptian Environmental Affairs Agency (EEAA). Environmental Guidelines for Municipal Solid Waste Landfill Site Selection. (2005).

[27] Hassaan, M. A. A GIS-Based Suitability Analysis for Siting a Solid Waste Incineration Power Plant in an Urban Area Case Study: Alexandria Governorate, Egypt. *J. Geogr. Inf. Syst.* **07**, 643–657 (2015).

[28] Feyzi, S., Khanmohammadi, M., Abedinzadeh, N. & Aalipour, M. Multi- criteria decision analysis FANP based on GIS for siting municipal solid waste incineration power plant in the north of Iran. *Sustain. Cities Soc.* **47**, 101513 (2019).

[29] Shi, X. et al. Using spatial information technologies to select sites for biomass power plants: A case study in Guangdong Province, China. *Biomass Bioenergy*. **32**, 35–43 (2008).

[30] Effat, A., Hegazy, N. & H. & Mapping potential landfill sites for North Sinai cities using spatial multicriteria evaluation. *Egypt. J. Remote Sens. Space Sci.* **15**, 125–133 (2012).

[31] Aragonés-Beltrán, P., Pastor-Ferrando, J. P. & García-García, F. Pascual-Agulló, A. An Analytic Network Process approach for siting a municipal solid waste plant in the Metropolitan Area of Valencia (Spain). *J. Environ. Manage.* **91**, 1071–1086 (2010). 20080331 10.1016/j.jenvman.2009.12.007

[32] Barakat, A., Hilali, A., Baghdadi, M. E. & Touhami, F. Landfill site selection with GIS-based multi-criteria evaluation technique. A case study in Béni Mellal-Khouribga Region, Morocco. *Environ. Earth Sci.* **76**, 413 (2017).

[33] Chabuk, A. et al. Combining GIS Applications and Method of Multi-Criteria Decision-Making (AHP) for Landfill Siting in Al-Hashimiyah Qadhaa, Babylon, Iraq. *Sustainability* 9, 1932 (2017).

[34] Wind, Y. & Saaty, T. L. Marketing Applications of the Analytic Hierarchy Process. *Manag Sci.* **26**, 641–658 (1980).

[35] Saaty, T. L. Fundamentals of the Analytic Hierarchy Process. In *The Analytic Hierarchy Process in Natural Resource and Environmental Decision Making* Vol. 3 (eds Schmoldt, D. L. et al.) 15–35 (Springer Netherlands, 2001).

[36] Karim Ghani, W. A. W., Ab., Rusli, I. F., Biak, D. R. A. & Idris, A. An application of the theory of planned behaviour to study the influencing factors of participation in source separation of food waste. *Waste Manag*. **33**, 1276–1281 (2013). 23415709 10.1016/j.wasman.2012.09.019

[37] Fielding, A. H. & Bell, J. F. A review of methods for the assessment of prediction errors in conservation presence/absence models. *Environ. Conserv.* **24**, 38–49 (1997).

[38] Wang, D., Tang, Y. T., He, J., Yang, F. & Robinson, D. Generalized models to predict the lower heating value (LHV) of municipal solid waste (MSW). *Energy* **216**, 119279 (2021).

[39] Egyptian Meteorological Authority (EMA). *Annual Meteorological Report: Wind Directions and Speed in Upper Egypt (Beni Suef Station)*. https://www.ema.gov.eg (2024).

[40] Yalcinkaya, S. & Kirtiloglu, O. S. Application of a geographic information system-based fuzzy analytic hierarchy process model to locate potential municipal solid waste incineration plant sites: A case study of Izmir Metropolitan Municipality. *Waste Manag Res. J. Sustain. Circ. Econ.* **39**, 174–184 (2021). 10.1177/0734242X20939636 32662341

[41] Chullamon, V. GIS-based Site Analysis for Selecting Suitable Sitesof Waste-to-energy Plants in Pathumthani. *Suan Sunandha Sci. Technol. J.* 7, 23 to 29 (2020).

